# Stepwise evolution and convergent recombination underlie the global dissemination of carbapenemase-producing *Escherichia coli*

**DOI:** 10.1186/s13073-019-0699-6

**Published:** 2020-01-20

**Authors:** Rafael Patiño-Navarrete, Isabelle Rosinski-Chupin, Nicolas Cabanel, Lauraine Gauthier, Julie Takissian, Jean-Yves Madec, Monzer Hamze, Remy A. Bonnin, Thierry Naas, Philippe Glaser

**Affiliations:** 1 Unité EERA, Institut Pasteur, APHP, Université Paris Saclay, 28 Rue du Dr Roux, 75015 Paris, France; 2 UMR3525, CNRS, 28 rue du Dr Roux, 75015 Paris, France; 3 EA7361 Faculty of Medicine of University Paris-Sud, Le Kremlin-Bicêtre, France; 4 Department of Bacteriology-Hygiene, Bicêtre Hospital, APHP, Le Kremlin-Bicêtre, France; 5 Associated French National Reference Center for Antibiotic Resistance, Le Kremlin-Bicêtre, France; 6 Université de Lyon - Agence Nationale de Sécurité Sanitaire (ANSES), Unité Antibiorésistance et Virulence Bactériennes, Lyon, France; 7 Laboratoire Microbiologie Santé et Environnement (LMSE), Ecole Doctorale des Sciences et de Technologie, Faculté de Santé Publique, Université Libanaise, Tripoli, Lebanon

**Keywords:** Carbapenems, Bacterial evolution, Lateral gene transfers, Multidrug resistance, Porin, Penicillin-binding proteins

## Abstract

**Background:**

Carbapenem-resistant *Enterobacteriaceae* are considered by WHO as “critical” priority pathogens for which novel antibiotics are urgently needed. The dissemination of carbapenemase-producing *Escherichia coli* (CP-*Ec*) in the community is a major public health concern. However, the global molecular epidemiology of CP-*Ec* isolates remains largely unknown as well as factors contributing to the acquisition of carbapenemase genes.

**Methods:**

We first analyzed the whole-genome sequence and the evolution of the *E. coli* sequence type (ST) 410 and its disseminated clade expressing the carbapenemase OXA-181. We reconstructed the phylogeny of 19 *E. coli* ST enriched in CP-*Ec* and corresponding to a total of 2026 non-redundant isolates. Using the EpiCs software, we determined the significance of the association between specific mutations and the acquisition of a carbapenemase gene and the most probable order of events. The impact of the identified mutations was assessed experimentally by genetic manipulations and phenotypic testing.

**Results:**

In 13 of the studied STs, acquisition of carbapenemase genes occurred in multidrug-resistant lineages characterized by a combination of mutations in *ftsI* encoding the penicillin-binding protein 3 and in the porin genes *ompC* and *ompF*. Mutated *ftsI* genes and a specific *ompC* allele related to that from ST38 inducing reduced susceptibility to diverse β-lactams spread across the species by recombination. We showed that these mutations precede in most cases the acquisition of a carbapenemase gene. The *ompC* allele from ST38 might have contributed to the selection of CP-*Ec* disseminated lineages within this ST. On the other hand, in the pandemic ST131 lineage, CP-*Ec* were not associated with mutations in *ompC* or *ftsI* and show no signs of dissemination.

**Conclusions:**

Lineages of CP-*Ec* have started to disseminate globally. However, their selection is a multistep process involving mutations, recombination, acquisition of antibiotic resistance genes, and selection by β-lactams from diverse families. This process did not yet occur in the high-risk lineage ST131.

## Background

Antibiotic resistance is one of the most urgent public health concerns. The increasing rate in antimicrobial resistances worldwide suggests a bleak outlook in terms of morbidity, mortality, and economic loss [1]. Carbapenems are one of the last resort antibiotics used to treat infections caused by multidrug-resistant (MDR) Gram-negative bacteria [2]. Dissemination of carbapenem-resistant *Enterobacteriaceae* (CRE) threatens the efficacy of current treatment options. Carbapenem resistance may result from a combination of mutations leading to reduced permeability (e.g., porin deficiency) and overexpression of an extended-spectrum β-lactamase (ESBL) or a cephalosporinase that shows a weak activity against carbapenems [3]. However, the main resistance mechanism is the acquisition of a carbapenemase gene [4]. The major carbapenemases encountered in *Enterobacteriaceae* belong to Ambler class A (KPC-type), class B (metallo-β-lactamases IMP, VIM- and NDM-types), or class D (OXA-48-like enzymes) [5]. As these carbapenemases are now frequently encountered in *Escherichia coli*, carbapenemase-producing *E. coli* (CP-*Ec*) might follow the same expansion and dissemination in hospitals and the community as the one observed for CTX-M-type ESBL-producing *E. coli* isolates [6, 7], a scenario feared by public health authorities. This is especially worrisome as these isolates are usually resistant to multiple antibiotics.

The epidemiology of CP-*Ec* is complex with geographical diversity in terms of carbapenemase genes and of dominant lineages [4]. Most studies performed at national or hospital levels point to a broad diversity of isolates as defined by multilocus sequence typing, with some isolates belonging to a few dominant sequence types (STs) like ST38, clonal complex (CC) 10 (ST10, ST167, ST617), ST101, ST131, and ST410 that carry different carbapenemase genes [4, 8–14]. However, their prevalence varies significantly worldwide. Analysis of CP-*Ec* strains isolated in 16 countries between 2008 and 2013 revealed that 36% belonged to the pandemic ST131, which has driven the global spread of CTX-M-15 ESBL in *E. coli* [11]. Similarly, a survey of CRE strains in China showed that ST131 represented 34% of the isolates and ST167 17% [14]. But only a single ST131 isolate out of 140 CP-*Ec* was identified by the French National Reference Centre (Fr-NRC) between 2012 and 2013 [8]. Recently, the phylogenetic analysis of a Danish collection of ST410 isolates combined to an international set of isolates revealed a globally disseminated *Ec* clone carrying *bla*_OXA-181_ on an IncX3 plasmid. This lineage was predicted by a Bayesian analysis to have acquired *bla*_OXA-181_ around 2003 and subsequently *bla*_NDM-5_ around 2014 [13].

Despite public health implications, factors contributing to the emergence and the dissemination of CP-*Ec* lineages have not been explored. Here, by using an in-depth evolutionary and functional analysis of *Ec* ST410 and by extending it to the whole *E. coli* species, we show that acquisition of carbapenemase genes followed different evolutionary trajectories. In most STs, it occurred preferentially in specific disseminated lineages mutated in *ftsI* encoding penicillin-binding protein 3 (PBP3) and/or in the *ompC* and *ompF* porin genes. We also show that these mutations lead to a reduced susceptibility to some β-lactams including ertapenem. In phylogroup D and in particular in ST38, a specific *ompC* allele might explain the high prevalence of CP-*Ec* isolates within this lineage. On the other hand, we did not identify mutations in *ftsI* and *ompC* among ST131 isolates. These new data on the evolution of CP-*Ec* allow us to propose a model for their selection and dissemination.

## Methods

### Bacterial isolates, growth conditions, and antibiotic susceptibility testing

Features of the clinical *E. coli* isolates analyzed in this work are listed in Additional file 1: Table S1. Fifty *Ec* ST410 isolates came from the strain collection of the Fr-NRC for antibiotic resistance. Four *Ec* ST410 clinical isolates came from the microbiological collection of the Public Health Faculty of the Lebanese University (Tripoli, Lebanon) and three ST410 isolates of animal origin from the ANSES strain collection. Test for OmpC permeability to β-lactams was performed in a W3110 derivative strain deleted for *ompC* and *ompF* genes [15]. Antibiotic susceptibility was performed by the disk diffusion method following the Clinical & Laboratory Standards Institute (CLSI) guidelines [16] or by Etest (Biomérieux) following the manufacturer’s recommendations. For W3110 Δ*ompC* Δ*ompF* pOXA-232 strains carrying pACYC184 derivatives, disk diffusion assays were performed on Mueller Hinton (MH) agar plates supplemented with 2 mg/l chloramphenicol. Fitness was determined by growth curve analysis with an automatic spectrophotometer Tecan Infinite M200 during 24 h in LB, MH, or M9 media supplemented with 0.4% glucose. Growth metrics were estimated with the R package “growthcurver” [17]. The area under the curve which includes contributions of the most important growth parameters (log phase, growth rate, and carrying capacity) was used as a growth metric.

### Genome sequencing and genome sequences retrieved from sequence databases

*Ec* genomes were sequenced by using the Illumina HiSeq2500 platform, with 100 nucleotide (nt) single-end reads for the four isolates from Lebanon and 100 nt paired-end reads for the other isolates. Libraries were constructed by using the Nextera XT kit (Illumina) following the manufacturer’s instructions. The OXA-181-producing *Ec*-MAD ST410 isolate was selected as a reference strain and sequenced to completion by using the long-read PacBio technology; 10,947 *E. coli* and 1451 *Shigella* genome sequences deposited in the NCBI database (19 June 2018) were retrieved for a global analysis of the specificity of CP-*Ec* (Additional file 2: Table S2 for STs with at least 1 CP-*Ec* isolate or 1 isolate with a 4-AA insertion in FtsI). Ninety-six additional *Ec* ST167 isolates were retrieved from Enterobase (https://enterobase.warwick.ac.uk/). Raw reads from 62 *Ec* ST410 and 21 *Ec* ST38 isolates identified in Enterobase were retrieved from the NCBI database. (Additional file 2: Table S2). Redundancy in the genome collection was removed by filtering for isolates from the same study, diverging by less than 7 SNPs. We kept 1 randomly selected isolate. In case of differences in resistome, assuming that antibiotic resistance gene (ARG) loss was more likely than ARG gain, we kept an isolate with the largest number of ARG. Raw reads from the isolates of the major STs were retrieved from the NCBI database. When raw data were not available, raw reads were simulated from the genome assembly with ART [18].

### Sequence assembly, genome annotation, and mutation identification

The PacBio reads were assembled with the RS_HGAP_Assembly.3 protocol from the SMRT analysis toolkit v2.3 [19] and with Canu [20]. The consensus sequence was polished with Quiver [19] and manually corrected by mapping Illumina reads by using breseq [21]. Illumina-sequenced isolates were assembled with SPAdes [22], and the quality of the assemblies was assessed with Quast [23]. Contigs shorter than 500 bp were filtered out. All assemblies and downloaded genomes were annotated with Prokka [24]. The presence of antibiotic resistance genes and plasmid replicons was assessed with ResFinder [25] and PlasmidFinder [26], respectively. ResFinder and PlasmidFinder were run in local from scripts and databases downloaded from the repositories of the Center for Genomic Epidemiology (https://bitbucket.org/genomicepidemiology/). Graphs of genomic regions of interest were drawn with genoplotR [27]. For each ST analyzed (Warwick scheme), the pangenome was characterized with Roary [28], and the amino acid (AA) sequences of OmpC, OmpF, GyrA, ParC, and FtsI were identified from the orthologous table generated by Roary using default parameters. In the case of OmpC, we observed different allelic versions, which were clustered using cd-hit [29], with an AA sequence identity threshold of 0.95. AA sequences for GyrA and ParC were aligned with the mafft L-INS-i approach [30], and the AA changes at the quinolone resistance-determining region (QRDR) positions (positions 83 and 87, and 80 and 84 for GyrA and ParC, respectively) were identified with a customized Perl script.

#### Mapping, variant calling, and identification of SNPs of interest

Sequence reads were mapped to reference genomes with BWA [31]. For each of the analyzed STs, an isolate with a complete chromosome sequence was chosen. For ST361 and ST206, no strain with a complete genome sequence was available from public databases, and we selected the isolate with the smallest number of contigs. A pseudo-chromosome was generated after sorting the contigs with mauve [32] and used as a reference. Variant calling was performed with the Genome Analysis Toolkit v 3.6.0 [33]. The criteria for variants were occurrence of the alternative base in more than 90% of the reads covering the position, a depth coverage of at least 10 (DP > 10), a quality by depth (QD) > 2, a Fisher strand bias (FS) < 60, a mapping quality (MQ) > 40, a mapping quality rank-sum test (MQRankSum) > − 12.5, and a read position rank-sum test (ReadPosRankSum) > − 8. For *Ec* ST410 isolates, variants associated with different clades of the phylogeny were extracted with VCFtools [34] and annotated with snpEff [35]. The effect of the non-synonymous mutations was assessed with the sorting intolerant from tolerant (SIFT) algorithm [36]. The algorithm searches for protein homologs in the refseq database using mutated proteins as query and assigns a score to each position. This score is weighted by the properties of the AA changed. If this score is below a threshold (0.05), the change is predicted to be functional.

#### Recombination detection and phylogenetic reconstruction

Pseudo-chromosomes were generated for each strain by parsing the pileup files created with SAMtools [37] using a Perl script. Constant positions relative to the reference sequence were called as such, and the alternative base was called if the polymorphic site had passed the SNP filters described above. A non-mapped position was called as a gap. Recombined regions were detected by using Gubbins v2.3.4 [38]. Maximum likelihood phylogenies were built for each ST by using the core non-recombinant SNPs identified in the variant calling step. Each tree was estimated with RAxML v8.2.8 [39] under the general time reversible (GTR) substitution model with a gamma-distributed rate over sites and an ascertainment bias correction. The maximum likelihood phylogeny of OmpC protein sequences was inferred with RAxML [39]. OmpC protein sequences were aligned with the mafft L-INS-i approach [30]. Gblocks [40] was used to refine the alignment, and the best fit model (WAG, with a gamma distribution) was estimated with protest 3 [41]. The visual display of phylogenetic trees was done with FigTree (http://tree.bio.ed.ac.uk/software/figtree/) and annotated trees with the script plotTree, R (https://github.com/katholt/plotTree).

### Testing the independence between mutations in *ftsI*, *ompF*, and *ompC* and the acquisition of carbapenemase genes

To assess the association between the different genetic events, we used the method developed by Behdenna et al. [42] implemented in the software EpiCs. The described events were mapped on the tree by parsimony, and the probability distribution of the number of paired events occurring in the tree was computed under the null model of independence. Two types of paired events are described in the following methods: inseparable pairs, when both events occur in the same branch, and genealogically ordered pairs, when the second event is found in a node more recent than the first one. We have considered the following genetic events: (i) “*ompC* mutations” encompassing acquisition of *ompC* alleles from phylogroup D strains by recombination, *ompC* mutations changing the charge of AA in the pore lumen, and *ompC* inactivation; (ii) “*ompF* mutations” including *ompF* gene inactivation and point mutations in the OmpR binding sites of its promoter; (iii) “*ftsI* mutations” including the four different four-codon insertions (YRIN, YRIK, TYPI, and YTIP) in *ftsI*; and (iv) the acquisition of a carbapenemase gene. We have focused our analysis on the independence between carbapenemase gene acquisition and mutation in each of the three genes, *ompC*, *ompF*, and *ftsI*.

### Complementation of the W3110 Δ*ompC* Δ*ompF* strain

*ompC* alleles and their regulatory regions were cloned into the medium copy number vector pACYC184 [43] following amplification by primers *ompC*_*Xba*_F and *ompC*_*Hind*_R (Additional file 3: Table S3), digestion by *Xba*I and *Hind*III restriction enzymes, and ligation into the vector digested by the same enzymes. Ligation was transformed into commercial *E. coli* TOP10 competent cells (Invitrogen). The absence of mutation was checked by Sanger sequencing. Five different alleles were cloned into pACY184: wild-type (WT) MG1655 and the G137D mutated allele, the ST410 WT allele and the R195L mutated allele from *Ec-*MAD and the ST38 allele. Plasmids containing the *ompC* genes as well as the empty vector were introduced into competent W3110 Δ*ompC* Δ*ompF* pOXA-232. Competent cells were prepared by the CaCl_2_ method [44]. Plasmid pOXA-232 [45] was prepared from an ST231 *Klebsiella pneumoniae* isolate from the Bicêtre Hospital collection carrying this plasmid. Plasmid content in transformants was verified by plasmid DNA extraction (Qiagen) and Sanger sequencing.

### Construction of *ftsI* mutant strains

The three mutations identified in the *ftsI* gene of the MAD strain were reconstructed in a MG1655 genetic background to analyze their effects on antibiotic resistance. To this aim, we introduced the 12 nt insertion (YRIN form) and the 2 non-synonymous SNPs (E349K and I532L) into the *E. coli* strain MGF (MG1655strepR_F’tet_∆traD::Apra) by TM-MAGE [46]. Briefly, an overnight culture of strain MGF transformed by pMA7SacB was used to inoculate 5 ml LB medium supplemented with tetracycline (7.5 mg/l) and carbenicillin (100 mg/l) (LB-TC) and grown at 37 °C until OD_600_ reached 0.6–0.7. The recombinase and Dam methylase were induced by the addition of l-arabinose (final concentration of 0.2% w/v) and further incubation for 10 min. Cultures were then chilled for 15 min on ice and centrifuged at 7300*g* at 4 °C. Two successive washes by 50 and 10 ml of cold water were performed, and the final pellet was resuspended in 200 μl water. One hundred microliters of cells was used for electroporation with 2 μl oligonucleotides Mut1*ftsI* or Mut2*ftsI* (Additional file 3: Table S3) alone or in combination at 20 μM each. The Mut1f*tsI* oligonucleotide carries both the 12-nt insertion and the E349K mutation, while the Mut2*ftsI* oligonucleotide has the I532L mutation. The content of the electroporation cuvette was used to inoculate 5 ml of LB-TC and submitted to three additional cycles of growth-induction-preparation of electrocompetent cells and electroporation. Following the last electroporation step, cells were resuspended in 1 ml LB and plated onto LB-TC agar plates. Mutations in isolated colonies were tested by PCR using primers complementary to mutant or WT alleles (Additional file 3: Table S3). Mutated colonies were grown on plates containing 10 g/l tryptone, 5 g/l yeast extract, 15 g/l agar, and 5% w/v sucrose for plasmid curing. Mutant strains were sequenced by using Illumina MiSeq platform, with 150-nt paired-end reads and Nextera XT kit (Illumina) for library preparation. Reads were mapped onto the MG1655 genome (Genbank NC_000913.3) to confirm that mutations in *ftsI* gene have been correctly introduced and to check that the rare off-target mutations are not predicted to interfere with the β-lactam susceptibility phenotype (Additional file 3: Table S4).

### RNA extraction and quantitative RT-PCR

Bacteria were grown in LB medium until OD_600_ reached 0.30–0.33. Ten microliters of culture was supplemented with 0.3 M final concentration of sodium chloride (NaCl) or with the same volume of water as control and further incubated for 20 min. Bacterial pellets were collected and stored at − 80 °C. Total RNA was extracted with the Total RNA purification kit Norgen Biotek. cDNAs were synthetized from 500 ng of RNA with the Superscript II reverse transcriptase (Invitrogen, Life Technologies). Primer pairs were designed for the *ompC* and *ompF* genes, targeting divergent regions from these two genes, and for the reference gene *recA* (Additional file 3: Table S3). The SYBR Green PCR kit (Applied Biosystems, Life Technologies) was used to perform quantitative PCR, and the relative expression of porin genes was measured by a standard curve method where the regression analysis was performed from serial dilutions of a mixture of control cDNAs. The expression value of each gene was normalized against the expression of the housekeeping gene *recA*. Each point was measured in triplicate, and three independent cultures were used for each strain in each condition.

### Statistical analysis

The statistical significance of the differences in the expression in qRT-PCR experiments was assessed by using a two-tailed *t* test. The statistical significance of the differences in the number of ARG between bacterial groups in different STs was assessed using the Wilcoxon rank sum-test implemented in R (v3.4.4). One-sided test was used for the comparison of the number of ARG, and two-sided test was used for the comparison on the area under the growth curve between the six isolates from the ST410 fluoroquinolone-resistant (FQR) clade.

## Results

### Most CP-*Ec* ST410 isolates received by the French NRC belong to a single lineage

**Fig. 1:**
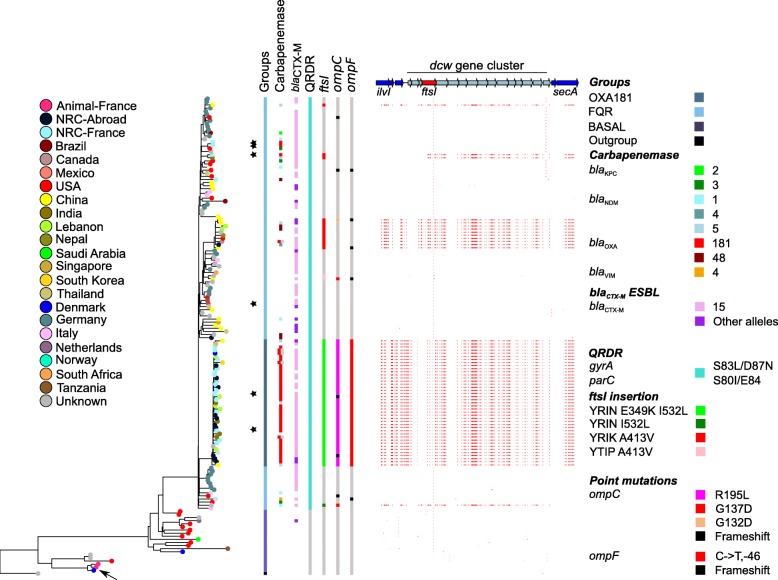
Core genome phylogeny and genomic features of *E. coli* ST410 isolates. ML phylogeny of 155 *Ec* ST410 nr genomes built with RAxML [39] based on the 3,937,051-bp core and recombination-free alignment of 3866 SNPs. The *Ec* ST88 isolate 789 (CP010315.1) was used as an outgroup. Isolates (branch tips) are color-coded according to the geographical origin as indicated in the figure key (left). Genomic features are indicated as indicated in the figure key (right) from left to right: groups according to the phylogeny, including the FQR clade and the OXA-181 subclade, carbapenemases, CTX-M ESBL, mutations in *gyrA* and *parC* QRDR region (FQ resistance); mutations in *ftsI*, *ompC*, and *ompF*. SNPs in the *dcw* cluster compared to the *Ec* ST410 non-recombined strain ANSES30599 (black arrow) are indicated by small vertical red bars. The upper part, genetic map of the *dcw* locus, genes are indicated by arrows, *ftsI* in red. NRC stands for National Reference Centre. Isolates analyzed for β-lactam susceptibility and fitness are indicated by stars (Fig. 7)

In order to determine the genetic bases for the dissemination of CP-*Ec* lineages, we first analyzed ST410 CP-*Ec* isolates, which show a high prevalence among isolates collected by the Fr-NRC [8]. We sequenced the genomes of 54 CP-*Ec* isolates, 50 collected by the Fr-NRC (including 22 from patients repatriated from 15 different countries), 4 from Lebanon, and 3 non-CP isolates of animal origin (Additional file 1: Table S1). We reconstructed their phylogeny together with 148 *Ec* ST410 genome sequences retrieved from public databases (Additional file 2: Table S2). We filtered for redundancy in this collection by removing 50 clonal isolates differing by less than 7 SNPs in the core genome [47] and keeping the isolate with the largest number of ARG. The phylogeny is in agreement with the recent analysis of CP-*Ec* ST410 from a Danish collection [13], with a major fluoroquinolone-resistant clade (FQR-clade) gathering the majority of the non-redundant (nr) isolates (133 out of 155) and of the nr isolates carrying carbapenemase genes (62 out of 63). Within the FQR-clade, 77% of the isolates carried CTX-M-type ESBLs (Fig. 1). Thirty-six out of the 40 *bla*_OXA-181_-carrying isolates formed a single subclade (the OXA-181 subclade) which corresponds to the previously described clade B4/H24RxC [13]. The 24 CP-*Ec* isolates not belonging to the OXA-181 subclade carry different carbapenemase genes from the OXA-48, KPC, VIM, and NDM families.

To accurately analyze the evolution of the OXA-181 subclade, we sequenced to completion a representative isolate of this clade (*Ec*-MAD). *Ec*-MAD carries 3 plasmids and 16 ARGs targeting 7 classes of antibiotics (Additional file 3: Table S5). Indeed, antibiotic susceptibility testing showed that it is resistant to most antibiotics tested, remaining susceptible only to imipenem, meropenem, doripenem, amikacin, azithromycin, chloramphenicol, tigecycline, and colistin and intermediate to mecillinam, ertapenem, kanamycin, and gentamicin (Additional file 3: Table S6). Comparison of the ARG content among ST410 *Ec* isolates revealed an increase in the median number of ARG between the basal isolates (*n* = 4), the FQR-clade (*n* = 9), and the OXA-181 subclade (*n* = 16) (Additional file 4: Figure S1).

### Gain of specific *ftsI* alleles by recombination is a hallmark of *Ec* ST410 carbapenemase-producing strains

Our phylogenetic analysis provided further evidence of a worldwide dissemination of the OXA-181 subclade [13]. Therefore, we searched for polymorphisms that, in addition to the acquisition of ARGs, have contributed to the expansion of this lineage. To this end, we systematically analyzed mutations occurring in the branch leading to its most recent common ancestor (MRCA). Besides 84 mutations in non-recombined regions, we also identified 1622 SNPs in regions predicted as recombined by using Gubbins [38] (Additional file 3: Table S7). Ninety-two percent occurred in a 124-kb DNA region between *yaaU* and *erpA* (Additional file 4: Figure S2). In contrast, this recombined region was almost identical to sequences found in four ST167 and eight ST617 isolates from CC10. Strikingly, all but one of these isolates carried a carbapenemase gene. Furthermore, analysis of ST410 CP-*Ec* isolates outside the OXA-181 subclade revealed four additional recombination events overlapping the 124-kb recombined region identified in the OXA-181 subclade (Fig. 1, Additional file 4: Figure S2). These recombination events affected a subclade of ten nr isolates of different geographical origins including five CP isolates carrying different carbapenemase genes; two closely related CP-*Ec* isolates, one from India (*bla*_NDM-5_) and one from the Fr-NRC (*bla*_OXA-181_); and isolated CP-*Ec* isolates (Fig. 1). The 16.5-kb region shared by the five recombined regions encompassed the *dcw* (division and cell wall) locus from *ftsI* to *secM* (Fig. 1). It encodes major functions in cell wall synthesis and cell division, including *ftsI* encoding PBP3, a target of diverse β-lactams [48]. Overall, 75% (47/63) of the nr CP-*Ec* ST410 isolates had recombined in the *dcw* region (Fig. 1).

One hundred ninety-seven SNPs including 16 non-synonymous (NS) mutations differentiated the common 16.5-kb region in the OXA-181 subclade from other *Ec* ST410 isolates (Additional file 3: Table S7). Among the differences, we identified an insertion of 4 codons (YRIN) between P333 and Y334 of *ftsI*. Strikingly, insertions of 4 codons at the same position in *ftsI* (YRIN in one case and YRIK in three) were also observed in the other ST410 isolates recombined in the *dcw* region. These insertions resulting from a 4-codon duplication (YRIN) and from a subsequent mutation (YRIK) were first described in NDM-producing *E. coli* isolates from different STs [49]. Additional NS SNPs were identified in the *ftsI* gene: E349K and I532L in association with the YRIN insertion and A413V with the YRIK insertion. The YRIK insertion in PBP3 was previously shown to confer reduced susceptibility to different β-lactams including ampicillin, cefepime, and aztreonam but not to carbapenems [49].

### Mutations in the porin genes *ompC* and *ompF* are predicted to also have contributed to the selection of the ST410 OXA-181 subclade

To identify additional polymorphisms that might have contributed to the dissemination of the *Ec* ST410 OXA-181 subclade, we analyzed the potential effect of non-synonymous mutations in the branch leading to its MRCA by using the SIFT algorithm [36]. We identified 34 NS SNPs with a predicted functional effect (9 in the recombined region) (Additional file 3: Table S8). Eight of these mutations affected genes from the class “transporter” including the multidrug efflux transporter components *emrD* and *emrK* and 5 from the class “cell envelope.” These mutations might have been selected in relation to modifications in antibiotic susceptibility.

**Fig. 2:**
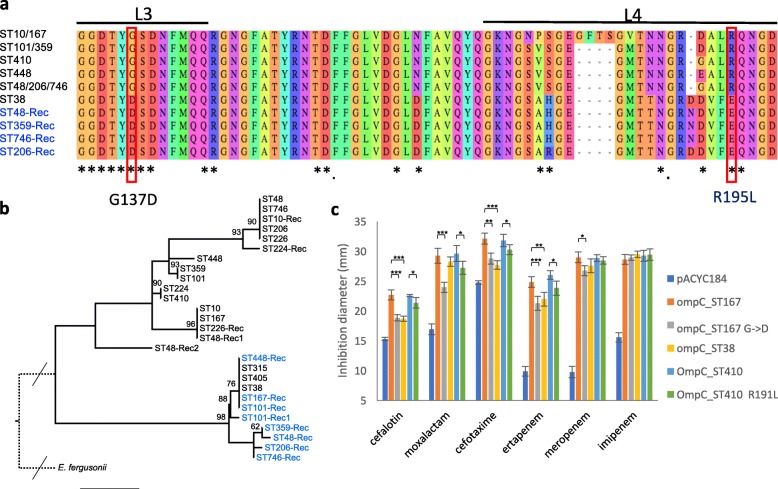
Mutations and recombination in the *ompC* gene. **a** Alignment of OmpC L3L4 region from ST in which mutations or recombination events were detected. Loops L3 and L4 are indicated by lines above the sequences, and those positions predicted to be exposed to the pore lumen in *E. coli* MG1655 (ST10) by asterisks [50]. Mutation R195L and G137D associated with the gain of carbapenemase genes are highlighted by red rectangles. Numbering is according to MG1655 OmpC protein. **b** Maximum likelihood phylogenetic reconstruction of representative OmpC sequences. OmpC sequences are labeled according to the ST of origin. OmpC sequences tagged with “-Rec” in blue were acquired by recombination in their respective STs; independent recombination events with different *ompC* alleles within a single ST are numbered. Bootstrap values > 60 are indicated. **c** Antibiotic susceptibility testing (diameters of inhibition) of W3110 Δ*ompC* Δ*ompF* pOXA-232 strain complemented by different alleles of the *ompC* gene cloned in the medium copy number pACYC184 [43] according to the figure key. The empty vector was used as control. Bars represent standard deviations; *P < 0.05; **P < 0.01; ***P < 0.001

**Fig. 3:**
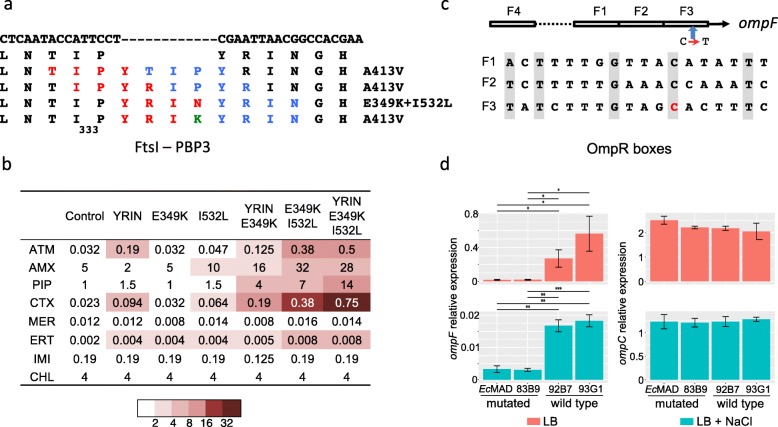
Functional analyses of *ftsI* and *ompF* mutations occurring in the OXA-181 *Ec* ST410 subclade. **a** Mutations identified in *ftsI*. The 4 different insertions following proline 333 resulted in the duplication of the 4 codons shown in red and blue. The YRIK insertion derived from YRIN by an N to K AA change (in green). The first and second lines represent the WT nucleotide and AA sequences, respectively; on the right, AA substitutions associated with each duplication. **b** Antibiotic susceptibility testing performed by Etest of MG1655 derivatives mutated in *ftsI*. Abbreviation: ATM, aztreonam; AMX, amoxicillin; PIP, piperacillin; CTX, cefotaxime; MER, meropenem; ERT, ertapenem; IMI, imipenem; CHL, chloramphenicol. Color code according to the figure key indicates the MIC ratio between each mutant and the reference strain (first column). **c** Schematic representation of the 4 OmpR binding sites in the *ompF* regulatory region and mutation of the conserved cytosine (C=>T) in the F3 OmpR binding site in red. **d** Expression of *ompF* and *ompC* genes in 2 strains from the OXA-181 subclade (*Ec-*MAD and 83B9, mutated) or from the FQR clade (92B7 and 93G1, WT) grown in LB medium and in LB medium supplemented with 0.3 M NaCl. Bars represent confidence intervals; *P < 0.05; **P < 0.01; ***P < 0.001

Among mutations affecting the functions related to the cell envelope, one was the *ftsI* mutation I532L; another affected the porin gene *ompC* at a conserved arginine residue in the L4 loop (R195L, OmpC MG1655 numbering), one of the gateways for carbapenems (Fig. 2a) [50]. Arg_195_ is exposed, at the vestibule of the pore lumen, and is conserved in OmpF [51]. Therefore, its replacement by leucine, a non-polar AA, might affect the permeation of β-lactams into the periplasm as we confirmed experimentally (see below). While we did not detect mutations in *ompF* coding sequence in the OXA-181 subclade, we identified a mutation in *ompF* regulatory region. This mutation replaces a conserved cytosine to a thymine residue in the proximal (F3) OmpR binding site. OmpR is a transcriptional activator of *ompF* and *ompC* expression, and this mutation is predicted to affect *ompF* expression (Fig. 3c) [52].

### Recombination at the *dcw* cluster and mutations in the porin genes *ompC* and *ompF* are frequently associated with the acquisition of a carbapenemase gene

In addition to the YRIN and YRIK insertions, 2 other 4-AA insertions were also previously reported at the same position in FtsI: YRIP and YTIP. These insertions result from duplications starting 2 and 3 codons upstream of the YRIN duplication, respectively (Fig. 3a) [53]. To determine whether the association between the acquisition of a carbapenemase gene and a mutated PBP3 characterized by a 4-AA insertion is specific to ST410 isolates or if it is also observed in other *E. coli* lineages, we analyzed *E. coli* and *Shigella* genomes from the NCBI database. None of the *Shigella* isolates encoded a carbapenemase gene or carried an insertion in PBP3; 487 *E. coli* isolates (4.4%) encoded a carbapenemase gene, and 248 (2.3%) carried a 4-AA insertion in PBP3: 163 YRIN, 49 YRIK, 3 YTIP, and 33 YRIP (Additional file 4: Figure S3, Additional file 5: Table S9). After removing redundancy for almost identical isolates of the same origin, 80% (146 out of 182) of nr isolates mutated in *ftsI* were CP-*Ec* (Additional file 5: Table S9). All the 123 nr isolates showing the YRIN insertion were also mutated at position 532 (I/L) and 112 at position 349 (E/K). On the other hand, all YRIK, YTIP, and YRIP insertions were associated with the same secondary mutation A413V (Fig. 3a), suggesting this AA change was selected together with the 4-AA insertion either to reduce the fitness cost of the AA insertion or to reduce the susceptibility to antibiotics targeting PBP3. Globally, these data reveal at the species level a strong link between these combinations of mutations in PBP3 and the acquisition of a carbapenemase gene. In addition to ST410, *ftsI* was mutated in the vast majority of the CP-*Ec* nr isolates from ST101 (100%, *N* = 23), ST167 (91%, *N* = 49), and ST405 (81%, *N* = 13) (Additional file 5: Table S9).

**Fig. 4:**
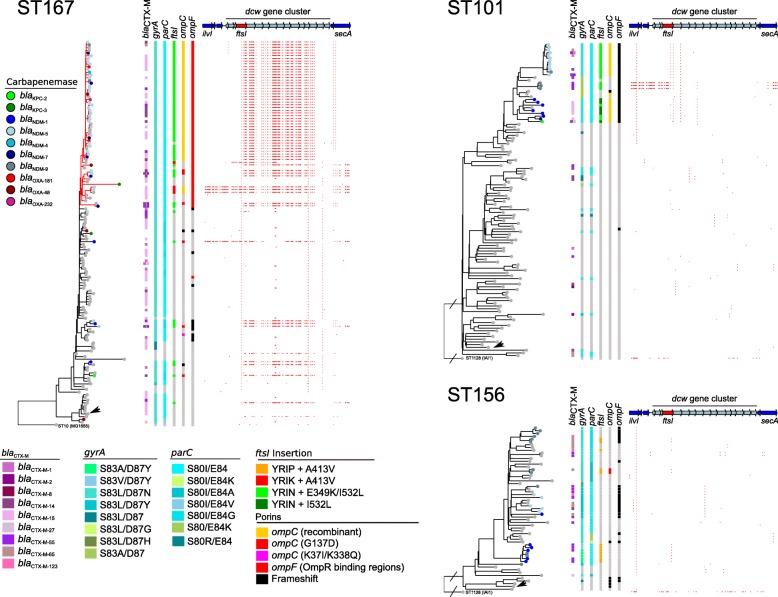
Phylogeny and mutations in non-redundant CP-*Ec* isolates of ST167, ST101, and ST156. ML phylogenies were estimated as for Fig. 1, using 4928, 17,146, and 13,481 non-recombinant SNPs for **a** *Ec* ST167, rooted with the ST10 strain MG1655 (NC_00913). The clade enriched in CP-*Ec* and defined by a mutation in an OmpR box within the *ompF* promoter region is indicated in red **b** *Ec* ST101 and **c** *Ec* ST156, respectively, both rooted with the ST1128 strain IAI1 (NC_011741). Branch tips indicate the presence and the type of carbapenemase according to the figure key on the left. On the right side of the tree, the following are represented from left to right: *bla*_CTX-M_ ESBL, *gyrA* and *parC QRDR* mutations, mutations in the *ftsI* gene, and genetic events affecting *ompF* and *ompC* according to the figure key at the bottom. SNPs in the *dcw* region are represented by small vertical red bars. Genes from the *dcw* locus are indicated by arrows and the *ftsI* gene in red. Black arrowheads indicate isolates used as a reference for SNPs mapping in the *dcw* gene cluster

In order to identify the most likely origin of these mutations, we reconstructed the phylogeny of STs with at least 1 isolate with a 4-codon insertion in *ftsI* and more than 5 isolates in total and analyzed SNPs using a basal strain of the ST as a reference. *ftsI* regions characterized by a higher density of SNPs than the rest of the genome were considered as possibly originating by LGT and recombination from a strain out of the ST as exemplified for ST167 (Fig. 4). Conversely, we speculated that in the isolates where the 4-codon duplications occurred or were vertically inherited, additional SNPs in the *ftsI* region would be rare. Indeed, we observed such a pattern for ST101 and ST156 strains (Fig. 4) and for 2 ST410 strains (Fig. 1) with YRIN, YRIP, and YTIP insertions, respectively. In contrast, all other *fts*I with a 4-codon insertion were in regions of higher SNP density indicating that they were acquired by recombination. The case of the *Ec* ST167 strains was particularly striking as shown in Fig. 4. We detected, within this single ST, after including 75 nr isolates from EnteroBase, 13 events of recombination distributed along the phylogeny of the FQR isolates and leading to the replacement of the endogenous PBP3 allele by an allele with the YRIN (*n* = 11) or the YRIK (*n* = 2) insertion. The recombined regions differed by their lengths and by their patterns of polymorphisms. This indicated that they were due to independent events occurring at different positions in the phylogeny. Eleven of these recombination events affected the ancestor of at least 1 CP isolate. In particular, the same recombined region was shared by a subclade of 40 nr isolates carrying carbapenemase genes from 7 different types, suggesting that recombination occurred in the ancestor of the subclade before the acquisition of a carbapenemase gene. Only 5 out of the 54 *Ec* ST167 CP isolates did not undergo recombination at *ftsI*. Strikingly, we also observed likely recombination events internal to ST101 and ST156 where the initial YRIN or YRIP insertions were predicted to have occurred. Indeed, the same combinations YRIN/L_532_ or YRIP/V_413_ were detected in scattered lineages of ST101 and ST156, respectively, suggesting intra-ST recombination events (Fig. 4).

By comparing the pattern of SNPs observed in the intra- and inter-ST alignments, we attempted to reconstruct recombination events across *Ec* phylogeny arising directly or indirectly from the original mutation events in ST101, ST156, and ST410. For instance, a 29.5-kb region was predicted to have recombined first from ST101 to ST167, and thereafter, a 124-kb region from ST167 might have recombined into the MRCA of the ST410 OXA-181 subclade (Additional file 4: Figure S4). Similarly, a 65-kb region with the YRIP insertion in *ftsI* from an ST156 *Ec* strain was likely introduced by homologous recombination into the MRCA of a clade of NDM-9 expressing ST224 *Ec* isolates (Additional file 4: Figure S4). Altogether, this suggests that mutations in *ftsI* have disseminated from ST101, ST156, and ST410 to other lineages by recombination. However, we cannot strictly rule out that the *ftsI* alleles might have recombined into the MDR lineages from multiple shared sources rather than from 1 MDR lineage to the next. The shortest recombination event, detected in a ST167 CP-*Ec* isolate carrying *bla*_NDM-5_ (WCHEC16), contains only the mutated *ftsI* gene with an YRIN insertion (Fig. 4). In total, we detected 52 independent recombination events involving a mutated *ftsI* allele scattered in all *E. coli* phylogroups except the B2 strains. Indeed, in *Ec* ST131 from the B2 phylogroup, despite a large number of CP-*Ec* isolates (*n* = 49 nr isolates), no isolate was mutated in *ftsI* (Additional file 4: Figure S5).

To determine whether mutations in *ompC* and *ompF* could similarly be associated with the acquisition of a carbapenemase gene, we reconstructed the phylogeny of STs with at least 3 CP-*Ec* isolates and then identified mutations that occurred in *ompC* and *ompF* during the evolution of these STs (Additional file 5: Table S9). We focused on mutations inactivating *ompF* or decreasing its expression by affecting the OmpR binding sites in the promoter region, as observed in the ST410 OXA-181 subclade. We also looked at mutations inactivating *ompC* or predicted to modify the porin permeability to β-lactams by decreasing the charge of AA located in the pore lumen [50]. One hundred seventeen CP-*Ec* nr isolates (41%) out of 286 from the NCBI were mutated in *ompF* compared to only 138 (8%) out of the 1659 non-CP-*Ec* nr isolates. This reveals a likely association between *ompF* alterations and acquisition of a carbapenemase gene. In 89 CP-*Ec* nr isolates (31%), OmpC was modified, but in only 3 CP-*Ec* (1%) isolates, it was inactivated. In non-CP-*Ec* isolates, OmpC was modified in only 44 (3%) nr isolates and inactivated or missing in 39 (2%) (Additional file 5: Table S9). Therefore, OmpC modifications, but not its inactivation, might also be associated with the acquisition of a carbapenemase gene. This might be due to the high fitness cost of OmpC loss [54]. In addition to the R195L mutation in the OXA-181 ST410 subclade, we identified two positions in the constriction loop L3 of OmpC [51] independently mutated in different isolates. The G137D replacement was identified in a ST361 lineage enriched in CP-*Ec* isolates (Additional file4: Figure S4) and in 4 independent CP-*Ec* isolates from ST410, ST448 and ST617 (Fig. 1 and Additional file 4: Figure S6), and the G132D in a carbapenemase-resistant isolate belonging to a ST410 lineage mutated in *ftsI* and in 2 ST405 isolates (Fig. 1 and Additional file 3: Figure S7). However, the most frequent *ompC* modification associated with CP-*Ec* isolates was the replacement of the original allele by alleles originating from phylogroup D strains through recombination (Fig. 2b). Indeed, we observed 20 independent recombination events, notably in the broadly distributed ST167 subcluster with a 22.7-kb recombined region from ST38 (Additional file 4: Figure S4). Strikingly, OmpC proteins from phylogroup D isolates differ from other *E. coli* OmpC proteins at the 2 aforementioned residues G137 and R195 by negatively charged residues, D and E, respectively (Fig. 2). In addition to ST38 [8, 9], 4 other STs from phylogroup D: ST354, ST405, ST457, and ST648 contained CP-*Ec* isolates (Additional file 4: Figure S7). The carriage of carbapenemase genes within these lineages points to an association between this *ompC* allele and the acquisition of the resistance gene.

### Acquisition of carbapenemase genes was preferentially selected in backgrounds mutated in *ompC*, *ompF*, and *ftsI*

**Fig. 5:**
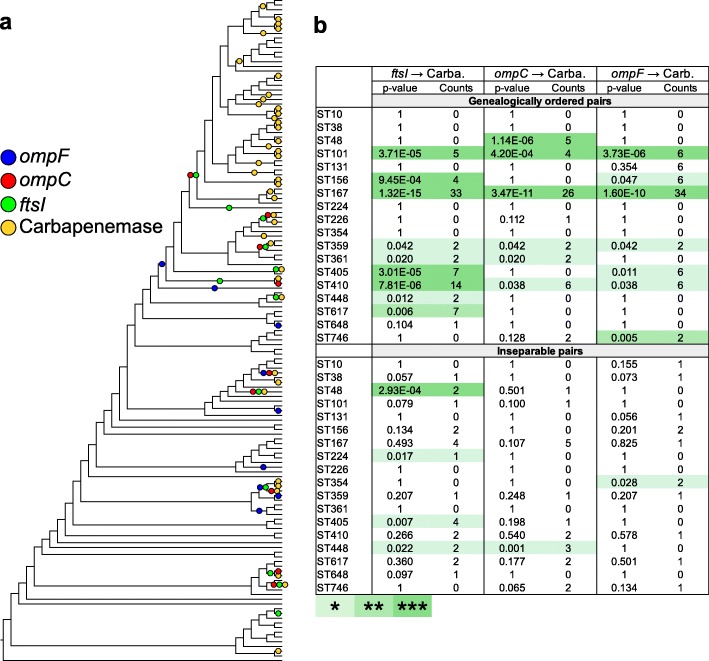
Test for the independence of the acquisition of carbapenemase alleles in defined genetic backgrounds. **a** Cladogram obtained from the maximum likelihood tree estimated for the ST167 *E. coli* isolates (Fig. 4). Four different genetic events, represented by colored circles, have been placed by parsimony on the tree. In blue, mutations affecting the *ompF* gene (inactivation, by frameshift of premature stop codons, and mutations in the regulatory region); in red, genetic events affecting the *ompC* gene (gains of new *ompC* alleles by homologous recombination, inactivation of the gene, and non-synonymous mutations modifying the charge of AA localized in the pore lumen); in green, homologous recombination of *ftsI* alleles with a four-codon insertion; and in yellow, acquisition of a carbapenemase gene. **b** Testing for the independence between the acquisition of carbapenemase genes and mutations in porin genes and/or *ftsI*. *P < 0.05; **P < 0.01; ***P < 0.001

The observation of frequent co-occurrences of mutations in the 3 genes and acquisition of a carbapenemase gene is indicative of a genetic association between these events. In order to statistically test for the association of 2 events in the phylogeny of each ST, we applied the method (EpiCs) developed by Behdenna et al. [42]. This method takes into account the topology of the tree and the node at which each event is predicted, by parsimony, to have occurred (Fig. 5a). The test is based on a probabilistic framework that computes the exact probability of counts of co-occurrences (2 events in the same branch) or subsequent events (1 preceding the other in the tree). This statistical analysis was repeated on STs containing at least 4 CP-*Ec* isolates after removing redundancy (Fig. 5b). In each case, both models, mutations occurring first or carbapenemase gene being acquired first, were tested. We obtained no evidence for the model where a carbapenemase gene was acquired first. In contrast, in 11 STs, a significant association was observed for mutations in *ftsI* and the acquisition of a carbapenemase gene, with the mutation predicted to have occurred first in nine ST. Similarly, *ompC* and *ompF* mutations show a significant association with carbapenemase acquisition in 7 and 8 STs, respectively, and were predicted to have occurred first in 6 and 7 STs, respectively. In 4 STs, mutations in the 3 genes precede the acquisition of the carbapenemase gene: ST167, ST101, ST359, and ST410. In total, the analysis showed that, within 13 STs, carbapenemase genes were preferentially acquired in a genetic background with a reduced susceptibility to β-lactams resulting from mutations in *ftsI*, *ompC*, or *ompF*.

We did not detect such an association for ST131 (49 nr-CP-*Ec* isolates), ST10 (12 nr-CP-*Ec* isolates), ST648 (11 nr-CP-*Ec* isolates), ST226 (7 nr-CP-*Ec* isolates), ST38 (26 nr-CP-*Ec* isolates), and ST69 (4 nr-CP-*Ec* isolates). ST648 and ST38 belong to phylogroup D, the phylogroup that served as the source for the dissemination of a specific *ompC* allele by recombination in other CP-*Ec* lineages. As this allele was present in the ancestors of the 2 STs and did not result from mutation or recombination during the evolution of the ST, it was not considered in our association analysis although it might confer a predisposition to acquire a carbapenemase gene. ST10 was the most numerous ST analyzed in this study with 528 nr isolates and showed a low rate of CP-*Ec* of 2% (Additional file 4: Figure S8). Despite the large number of CP-*Ec* ST131 isolates, none showed a 4-codon insertion in *ftsI* or an AA change in OmpC predicted to decrease susceptibility. In addition, among the 29 ST131 isolates with an inactivated *ompF* gene, only 8 carry a carbapenemase gene. Furthermore, CP-*Ec* were equally distributed in the 4 ST131 lineages A, B, C1, and C2 (Additional file 4: Figure S5). Therefore, the acquisition of a carbapenemase gene in ST131 isolates might proceed according to a different path.

### Carbapenemase genes were more frequently acquired in MDR backgrounds

**Fig. 6:**
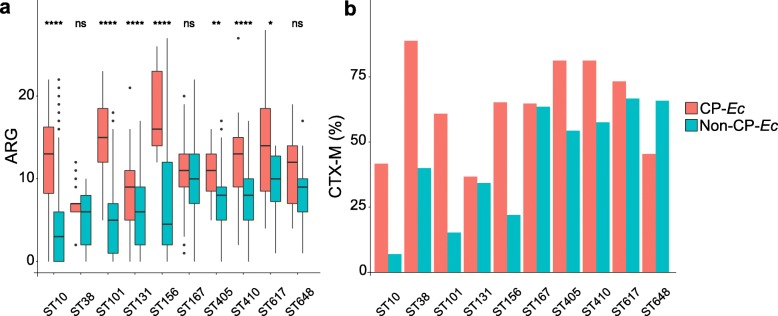
Occurrence of ARG and *bla*_CTX-M_ genes in CP-*Ec* isolates. **a** Comparison of the number of ARGs between CP-*Ec* and non-CP-*Ec* for the 10 STs encompassing more than 10 CP-Ec isolates. The horizontal lines in the boxes represent the median number of ARGs. The box boundaries represent the first and third quartiles of the distribution and box-plot whiskers span 1.5 times the interquartile range of the distribution. Outliers are denoted as black points outside the whiskers. Statistical significances were tested with a one-sided Wilcoxon rank-sum test. *P < 0.05; **P < 0.01; ****P < 0.0001; ns, non-significative. **b** Comparisons in % of the presence of *bla*_CTX-M_ genes between CP-*Ec* and non-CP-*Ec*

A characteristic of the ST410 OXA-181 subclade compared to other ST410 *Ec* isolates is a globally higher number of ARGs. To determine whether this observation can be extended to other CP-*Ec* isolates, we systematically analyzed for their ARG content the isolates belonging to the ten STs with more than ten CP-*Ec* isolates. In most STs, CP-*Ec* isolates showed a significantly higher number of ARG than non-CP *Ec* isolates. Only in ST38, ST167, and ST648 the number of ARG was not significantly superior in CP-*Ec* (Fig. 6). Note that both CP-*Ec* and non-CP-*Ec* ST167 isolates show a high number of ARG (median = 10). Similarly, we observed a higher percentage of CTX-M enzymes among CP-*Ec* isolates compared to non-CP-*Ec* isolates except in ST131 and ST648 (from phylogroup D).

### Mutations in *ftsI*, *ompC*, and *ompF* associated with CP-*Ec* contribute to decreased susceptibility to β-lactams

Our data show that specific mutations in *ompC*, *ompF*, and *ftsI* have been frequently selected in lineages that thereafter acquired carbapenemase genes by LGT. To further decipher the consequences of these mutations, we tested experimentally their impact on the susceptibility of *E. coli* to β-lactams. We first determined the contribution of the three mutations in *ftsI* (YRIN insertion, E349K, and I532L) identified in the OXA-181 lineage. To this aim, we constructed derivatives of the ST10 *s*train MG1655 with combinations of these mutations (Fig. 3b). Individually, each mutation showed only a small effect on susceptibility to β-lactam targeting PBP3. However, the combination of two or three mutations led to a stronger decrease in the susceptibility to these antibiotics. In particular, the MG1655 PBP3 derivative with the three modifications showed, in the absence of any β-lactamase, a 32-, 16-, and 14-fold increase in the MIC to the third-generation cephalosporin cefotaxime, to the monobactam aztreonam, and to piperacillin, respectively. This strain showed a slight increase in the MIC to ertapenem (× 4), which mainly targets PBP2 and to a lesser extent PBB3, but no difference in the MIC to meropenem and imipenem that show low affinity for PBP3 [55].

To test the impact of mutations and recombination in *ompC* on β-lactam permeability, we complemented an *E. coli* K12 strain lacking the two major porins and carrying pOXA-232 [45] and tested for susceptibility to β-lactams (Fig. 2c). The WT ST167 (CC10) *ompC* allele, and its G137D derivative; the ST38 (phylogroup D) allele; and the WT ST410 allele and its R195L derivative were cloned into the medium copy number vector pACYC184 [43]. Complementation with the different alleles of *ompC* led to an increased susceptibility to β-lactams tested. However, we observed a differential effect of the different *ompC* alleles (Fig. 2c). In particular, we observed that strains expressing the R195L, the G137D, and the ST38 *ompC* alleles showed reduced susceptibilities to cefalotin, cefoxitin, moxalactam, and ertapenem compared to the strain complemented with the WT ST167 and ST410 alleles. These results confirm our prediction that the two *ompC* variants and the ST38 allele associated with Cp-*Ec* isolates show lower permeability towards different β-lactams including ertapenem than their WT counterparts.

In *E. coli*, the OmpF porin has been shown to contribute to the permeation of β-lactams into the periplasm and to susceptibility to these antibiotics [56]. To estimate the impact on β-lactam susceptibility of the mutation in the *ompF* promoter region identified in the ST410 OXA-181 subclade, we quantified *ompF* mRNA by qRT-PCR. We compared *ompF* at normal and high osmolarity (LB and LB, 0.3 M NaCl) between two isolates from the OXA-181 subclade (mutated) and two isolates from the FQR clade (non-mutated). As a control, we also quantified *ompC* expression. We observed a 15 to 30 and 5-fold reduction in *ompF* expression in LB and LB-NaCl respectively in the mutated isolates compared to wild type, whereas *ompC* expression remained unchanged (Fig. 3d). This confirmed that the regulatory mutation identified in the OXA-181 subclade leads to a decreased *ompF* expression in these isolates that will reduce the entry of β-lactams into the periplasm and antibiotic susceptibility.

### Isolates of the OXA-181 subclade show higher resistance without in vitro fitness cost as compared to other OXA-181 *Ec* isolates of the FQR clade

**Fig. 7:**
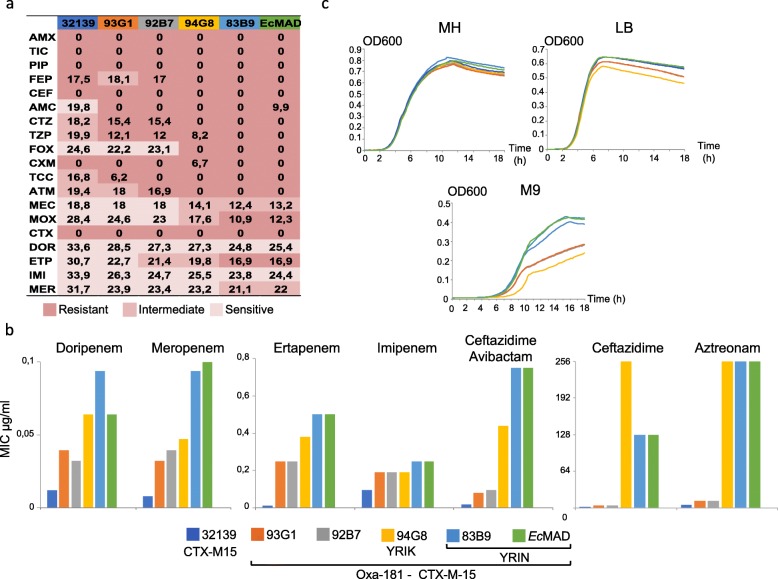
β-lactam susceptibility profiles and fitness of *Ec* ST410 strains. **a** β-lactam susceptibility determined by disk diffusion. Diameters are indicated in millimeters. Resistant, intermediate, and sensitive according to CLSI guidelines [16] are indicated by colors as defined in the figure keys; 32139 carries *bla*_CTX-M15_, 93G1 and 92B7 carry *bla*_CTX-M15_ and *bla*_OXA-181_, 94G8 carries *bla*_CTX-M15_ and *bla*_OXA-181_ and a mutated *ftsI* gene (YRIK), 83B9 and *Ec-*Mad belong to the OXA-181 subclade. Their position in the ST410 phylogenetic tree is indicated by stars in Fig. 1. **b** Minimal inhibitory concentrations (MIC) determined by Etest for selected β-lactams. **c** Growth curves in rich (LB and Müller Hinton, MH) and minimal (M9) medium. Curves represent the average value of 10 experiments. Box plot representations of the area under the curve of replicates determined by using growthcurver [17] are given in Additional file 4: Figure S9. Abbreviations: AMX, amoxicillin; TIC, ticarcillin; PIP, piperacillin; FEP, cefepime; CEF, cephalothin; AMC, amoxicillin-clavulanic acid; CTZ, ceftazidime; TZP, piperacillin-tazobactam; FOX, cefoxitine; CXM, cefuroxime; TCC, ticarcilline-clavulanic acid; ATM, aztreonam; MEC, mecillinam; MOX, moxalactam; CTX, cefotaxime; DOR, doripenem; ETP, ertapenem; IMI, imipenem; MER, meropenem. Antibiotic resistance gene repertoires of the 6 strains are given in Additional file 3: Table S10

Independently, mutations in *ftsI* and *ompC* affected the susceptibility to β-lactams of a laboratory strain. To determine the impact of mutations and ARG on β-lactam resistance and fitness in clinical ST410 isolates, we analyzed the phenotype of five *bla*_OXA-181_ *bla*_CTX-M-15_ *Ec* isolates from different lineages of the ST410 FQR clade (Additional file 3: Table S10) in comparison with an isolate expressing only CTX-M-15: two isolates from the OXA-181 subclade mutated in *ompC*, *ompF*, and *ftsI* (YRIN insertion), one isolate mutated in *ftsI* (YRIK insertion), and the two other isolates without any mutation in the three genes (indicated by stars in Fig. 1). We observed, by disk diffusion assay and Etest, a gradual decrease in the susceptibility to diverse β-lactams between the four groups of isolates: CTX-M15 < CTX-M15, OXA-181 < CTX-M15, OXA-181, and YRIK insertion in PBP3 < OXA-181 subclade (Fig. 7a, b). According to CLSI breakpoints, isolates from the OXA-181 subclade were resistant to almost all β-lactams tested except doripenem and imipenem and intermediate to meropenem and mecillinam. Higher resistance was partly due to the differences in β-lactamase gene content including *bla*_OXA-181_ (Additional file 3: Table S10). However, mutations in *ftsI*, including the YRIK and YRIN insertions, were likely responsible for the decreased susceptibility of ST410 *Ec* isolates to β-lactams targeting PBP3 like ceftazidime and aztreonam (Fig. 7b). The isolates from the OXA-181 subclade showed a decreased susceptibility to ertapenem and meropenem likely resulting from mutations in *ompC* and in *ftsI* for ertapenem.

To determine whether these mutations have an impact on fitness, we compared growth parameters in LB, MH, and M9 media as a proxy. Despite the higher resistance to antibiotics, we did not detect any significant differences in the growth parameters in rich medium among the 6 clinical isolates tested (Fig. 7c) suggesting these mutations have no fitness cost or their effect has been compensated by other mutations. The 2 isolates from the OXA-181 subclade and the non-OXA-181 isolate 32139 grew at a higher OD_600_ in the minimal medium than the 3 other isolates (Fig. 7c). Therefore, the high level of resistance to most β-lactams and the decreased susceptibility to carbapenems of isolates of the OXA-181 subclade do not seem to be at the expense of a lower in vitro fitness for the isolates we have analyzed.

## Discussion

During the past 20 years, the increased prevalence of ESBL-producing *Enterobacteriaceae* led to an increased use of carbapenems and selection of CP-*Ec* strains. The dissemination of CP-*Ec* lineages is particularly feared. The global dissemination of antibiotic-resistant clones and their evolutionary trajectory result from a trade-off between acquired resistance and biological cost in the absence of antibiotics [57]. However, how this trade-off is reached during in vivo evolution is largely unknown as it likely depends on multiple factors, such as antibiotic usage, which differs between areas of the world [58]. Also, clones may spread across various human and non-human sectors and successively undergo highly different selective pressures. In that context, both the surveillance of emerging Cp-*Ec* clones and their thorough genetic analysis are required to characterize their evolutionary trajectories and prevent the dissemination of other and possibly more virulent clones.

Here, we first analyzed the worldwide disseminated CP-*Ec* OXA-181 ST410 subclade and extended this analysis to the whole *E. coli* species. We showed that carbapenemase genes were preferentially acquired in lineages already mutated in 3 genes contributing to β-lactam resistance: *ompC*, *ompF*, and *ftsI*. Indeed, in 13 STs representing 54% (*n* = 234) of the non-redundant CP isolates analyzed in this work, a combined phylogenetic and statistical analysis revealed a significant association between mutations of these genes and subsequent acquisition of a carbapenemase gene (Fig. 5c). The association was best exemplified by a large clade among ST167 isolates first defined by a mutation in an OmpR box within the *ompF* promoter region (in red in Fig. 4). Within this clade, 7 events of recombination led to the acquisition of mutations in *ftsI* and 4 genetic events modified the *ompC* gene: 2 recombination events leading to its replacement by an allele from a phylogroup D strain and 2 homoplasic G137D mutations affecting AA of the pore lumen. Eventually, multiple events of acquisition of carbapenemase genes were selected (Fig. 4). Interestingly, the encoded carbapenemases belonged to NDM and OXA families that differ in the levels of carbapenem resistance and by the spectrum of β-lactams they hydrolyze [59]. Therefore, the scenario originally detected in the ST410 OXA-181 subclade expands to numerous other STs and does not seem to depend on the carbapenemase family.

Such a situation is reminiscent of what has recently been observed in the *K. pneumoniae* high-risk clones ST258, ST512, and ST11 [60]. In these clones, the acquisition of carbapenemase genes was frequently associated with the inactivation of the porin gene *ompK35* (equivalent of *E. coli ompF*) and mutations in *ompK36* (equivalent of *ompC*). In CP-*Ec*, we observed *ompC* inactivation in a few isolates, probably due to a high fitness cost of this event. Instead, the selected mutations affected *ompC* permeability towards β-lactams (Fig. 2c) while probably keeping the global function of the porin. This likely leads to a lower fitness cost than gene inactivation [54]. In contrast, *ompF* inactivation or mutations in its promoter region lowering transcription with likely a lower fitness cost were observed in CP-*Ec*. This indicates that, under in vivo growth conditions, the OmpF porin might be more easily dispensable than OmpC. On the other hand, PBP3 is essential for cell division, and selection of *ftsI* mutants is expected to be highly evolutionary constrained. This might explain the extremely rare *ftsI* mutations previously reported in clinical lineages of *E. coli*. This contrasts with the high frequency of *ftsI* mutations observed among Cp-*Ec* isolates.

During the evolution of CP-*Ec* lineages, recombination events involving *ftsI* and *ompC* were pervasive throughout the *E. coli* species except among phylogroup B2. We identified 4 combinations of mutations in *ftsI* frequently associated with CP-*Ec* isolates (Fig. 3a). To our knowledge, these modifications are the only mutations in *ftsI* contributing to β-lactam resistance reported in natural *E. coli* isolates [49, 53]. The chance of mutation combinations leading to a significant decrease in susceptibility is likely very low, but the selective advantage is strong. In agreement with this hypothesis, the phylogenetic reconstruction of the recombination events showed that these combinations arose likely only once and disseminated widely across the *E. coli* species by LGT, as we identified 52 events of recombination. Most of these recombination events were associated with at least 1 CP-*Ec* isolate (*n* = 46), and in 24 cases, it corresponded to disseminated lineages (i.e., with more than 3 isolates from different geographical origins). We observed a similar situation for the porin OmpC, with 20 events of recombination of *ompC* alleles originating from phylogroup D isolates. The chromosomal region next to the *ompC* gene has been shown to be a hotspot of recombination [61]. However, we observed that the acquisition of this specific allele was associated with the acquisition of carbapenemase genes in most cases (14 out of 20). Recombination has been shown to play a major role in β-lactam resistance in pneumococcus [62] or in *Neisseria* spp. [63]. Altogether, our data show for the first time that in addition to the LGT of β-lactamase genes located on MGE, recombination has a significant contribution to β-lactam resistance in *E. coli*, including carbapenems.

Further comparisons of β-lactam susceptibility and fitness among ST410 isolates carrying the same *bla*_OXA-181_-bearing plasmid and *bla*_CTX-M-15_ gene with different patterns of mutations in *ompC*, *ompF*, and *ftsI* showed that in these clinical isolates and particularly in the broadly disseminated OXA-181 subclade, the increased resistance to β-lactams was not associated with a fitness cost (Fig. 7c). We also observed that increased resistance to β-lactams could be attributable to mutations in the three genes, in agreement with our experimental study of these mutations individually (Figs. 2 and 3). In particular, for the OXA-181 subclade, we observed an additional decrease in the susceptibility to ertapenem. Interestingly, ertapenem shows a higher biliary excretion than other carbapenems and was found to have a stronger impact on the intestinal microflora [64]. *ftsI* mutations were also found to be selected during in vitro evolution in the presence of ertapenem but not meropenem [65]. However, other β-lactams like aztreonam might also have contributed to the selection of these combinations of mutations [66].

A systematic analysis of the number of ARGs across the *E. coli* species showed that a higher number of ARGs in CP-*Ec* compared to non-CP-*Ec* isolates were a common feature in all STs with more than 10 CP-*Ec* isolates. A single exception was ST167 where a similarly high number of ARGs was observed in both CP and non-CP-*Ec* isolates analyzed (Fig. 6). For these STs, the acquisition of carbapenemase genes occurred more frequently in an MDR background. We also observed a frequent co-occurrence of CTX-M family ESBL and carbapenemase genes in most STs, but not in the ST131 lineage.

In all, these data suggest a long-term and step by step evolution of those lineages with episodic periods of selection and dissemination. As a first step, specific mutations in *ompF*, *ompC*, or *ftsI* would have been fixed in isolates already carrying different β-lactamases genes including ESBL, leading to low levels of resistance to carbapenems [67]. In the second step, under antibiotic pressure, the combination of these mutations and β-lactamase expression might have favored the efficient conjugative transfer of plasmids carrying carbapenemase genes from other CP-bacterial species by increasing the proportion of donor and receptor bacteria [68]. This might have occurred in the context of low levels of carbapenems or other β-lactams, such as found in the gut during parenteral administration of antibiotics with biliary excretion. This model could also explain the high prevalence of ST38 isolates observed both in England and in France [8, 9], as 24 out of 27 (89%) ST38 CP-*Ec* isolates carry a CTX-M class gene.

Although CP-*Ec* were frequent among the ST131 isolates studied here, with 49 nr isolates (66 in total), we did not observe any case of four AA insertions in *ftsI* or of mutations affecting AA in the pore lumen of OmpC among the 402 nr genome sequences we have analyzed. Furthermore, CP-*Ec* isolates were broadly distributed among the different ST131 lineages and were not associated with CTX-M type ESBL since only 37% also carried a β-lactamase gene of this class (Fig. 6b)*.* The selection for ST131 CP-*Ec* isolates might therefore follow a different path compared to other CP-*Ec*, which might be related to their higher and human-specific pathogenicity [69]. ST131 CP-*Ec* isolates might arise sporadically in patients following conjugation of carbapenemase gene-carrying plasmids from another CPE and subsequent selection by β-lactams, including treatments with carbapenems. These transconjugants would be more frequently detected in clinics due to their high pathogenicity.

## Conclusions

Despite the clinical importance of carbapenemase-producing *E. coli*, factors contributing to their selection and their dissemination remain largely unknown. Here, by combining evolutionary and comparative genomics, we identified three different evolutionary trajectories associated with the gain of carbapenemase genes. In most STs with a high prevalence of CP-*Ec* isolates like ST410, ST167, or ST101, carbapenemase genes were preferentially acquired in backgrounds mutated in genes contributing to β-lactam decreased susceptibility and frequently carrying a larger number of ARGs, including ESBL genes of the *bla*_CTX-M_ family, than non-CP-*Ec*. This trajectory might result from a step by step selection following the use of β-lactams of different families including carbapenems. In ST38, the scenario would be similar with a larger repertoire of ARGs including *bla*_CTX-M_ among CP-*Ec* compared to non-CP-*Ec*. However, phylogroup D strains would be intrinsically less susceptible to β-lactams than other lineages due to a specific allele of the OmpC protein. On the other hand, ST131 CP-*Ec* isolates were neither associated with CTX-M ESBL genes or mutation in *ompC* and *ftsI*. Reassuringly, the selected ST131 CP-Ec isolates, possibly due to their lower fitness, have not yet disseminated globally. Indeed, despite the high prevalence of ST131 CP-*Ec* reported in different studies [11, 70, 71], there was no indication of global dissemination of a specific lineage among the ST131 CP-*Ec* genome sequences we have analyzed (Additional file 4: Figure S5). In most STs, the evolution of CP-*Ec* clones is more complex than the mere acquisition of a carbapenemase gene. Recombination and horizontal transfer of specific alleles of *ftsI* encoding PBP3 and of the porin gene *ompC* have a major contribution in shaping the genomes of strains which will preferentially acquire a carbapenemase gene. Therefore, besides the LGT of resistance genes, LGT of mutated genes from the core genome deserves to be followed up in surveillance programs of CP-*Ec*.

## Supplementary information

**Additional file 1: Table S1.** Characteristics of the *E. coli* isolates analysed in this work.

**Additional file 2: Table S2.** *E. coli* genome sequences retrieved from public databases.

**Additional file 3: Table S3.** Oligonucleotides used in this work. **Table S4.** Mutations detected in MG1655 strains derivatives mutated in *ftsI*. **Table S5.** Genomic features of the *E. coli* ST410 isolate *Ec-*MAD. **Table S6.** Antibiotics susceptibility testing of the ST410 isolate *Ec-*MAD. **Table S7.** Distribution and effect of point mutations in the OXA-181 *E. coli* ST410 subclade MRCA. **Table S8.** Mutations predicted with a functional effect using the SIFT algorithm. **Table S10.** Antibiotic resistance gene content of ST410 isolates analysed in Fig. 7.

**Additional file 4: Figure S1.** Number of antibiotic resistance genes (ARG) according to the phylogenetic group among *E. coli* ST410 isolates. **Figure S2.** Recombination events in isolates of the *E. coli* ST410 FQR clade. **Figure S3.** Phylogenetic distribution of *E. coli* isolates mutated in *ftsI*. **Figure S4.** Recombination events at the *dcw* and *ompC* loci. **Figure S5.** Phylogeny and mutations in non-redundant *E. coli* ST131 CP-*Ec* isolates. **Figure S6.** Phylogeny and mutations in non-redundant CP-*Ec* isolates of ST48, ST206, ST224, ST359, ST361, ST448 and ST617. **Figure S7.** Phylogeny and mutations in non-redundant *E. coli* isolates of phylogroup D. **Figure S8.** Phylogeny and mutations of ST10, ST226, and ST746 non-redundant *E. coli* isolates. **Figure S9.** Estimated fitness of *E. coli* ST410 strains in rich and minimal media.

**Additional file 5: Table S9.** *ftsI, ompC* and *ompF* mutations in STs encompassing CP-*Ec* isolates.

**Additional file 6:.** Source data file of the work.

## Data Availability

The datasets generated and/or analyzed during the current study are available as follows: Illumina reads from the 57 newly sequenced isolates and the complete genome assembly of strain Ec-MAD have been deposited in the EMBL nucleotide sequence database (http://www.ebi.ac.uk/ena) under study accession number PRJEB27293 [72] and PRJEB27274, respectively [73]. The accession numbers for individual isolates are listed in Additional file 1: Table S1. Source data of studies presented in Figs. 2, 3, and 6 and Additional file 4: Figures S1 and S9 are provided in the Additional file 6: Source data. Codes of custom scripts are available at https://github.com/rpatinonavarrete [74]. *E. coli* genomes listed in Additional file 2: Table S2 were retrieved from the following sequence libraries: NCBI *E. coli* Genome Assembly and Annotation report [https://www.ncbi.nlm.nih.gov/genome/?term=Escherichia%20coli] [75]. Enterobase *E. coli* database [http://enterobase.warwick.ac.uk/species/index/ecoli] [76].

## Abbreviations

AA: Amino acid
ARG: Antibiotic resistance gene
CRE: Carbapenem-resistant *Enterobacteriaceae*
CP-*Ec*: Carbapenemase-producing *E. coli*
CC: Clonal complex
*Ec*: *Escherichia coli*
ESBL: Extended-spectrum β-lactamase
Fr-NRC: French National Reference Centre
MRCA: Most recent common ancestor
MDR: Multidrug-resistant
nr: Non-redundant
NS: Non-synonymous
ST: Sequence type
WT: Wild type
