# Supplementary material for: Stepwise evolution and convergent recombination underlie the global dissemination of carbapenemase-producing Escherichia coli
Source: Genome Med. 2020 Jan 20;12:10. doi: 10.1186/s13073-019-0699-6 (PMC6970295; doi:10.1186/s13073-019-0699-6)
Supplement: Supplementary file 3 — Additional file 3: Table S3. Oligonucleotides used in this work. Table S4. Mutations detected in MG1655 strains derivatives mutated in ftsI. Table S5. Genomic features of the E. coli ST410 isolate Ec-MAD. Table S6. Antibiotics susceptibility testing of the ST410 isolate Ec-MAD. Table S7. Distribution and effect of point mutations in the OXA-181 E. coli ST410 subclade MRCA. Table S8. Mutations predicted with a functional effect using the SIFT algorithm. Table S10. Antibiotic resistance gene content of ST410 isolates analysed in Fig. 7. [file 13073_2019_699_MOESM3_ESM.pdf]

Table S3. Oligonucleotides used in this work

Gene	Name	Sequence (5'-3')	Experiment
ompC	ompC-F	GCGAAGGCATGACCAACAAC	qRT-PCR
ompC	ompC-R	AGGTCTGGGTGTACTGAGCA	qRT-PCR
ompF	ompF-F	ACCAACCGCAAGAAGCTCA	qRT-PCR
ompF	ompF-R	TTGGTGTAAGCGATGGACGG	qRT-PCR
recA	recA-F	GAAGACCGTTCCATGGATGT	qRT-PCR
recA	recA-R	GCGTGTTCAGCATCGATAAA	qRT-PCR
ftsI	Mut1ftsI	C*C*G*G*TCAGGGTTAATTTGCTGTA GCGTGCCACGTCTTTGATTTCGTGGCC GTTAATTCGATAGTTAATTCGATAAGG AATGGTATTGAGTA§	TM-MAGE
ftsI	Mut2ftsI	C*C*A*T*GATGGCACCAAAGACCGG CGCGGAAACGGCGCCGCCGTAGTATT TACCCGCCTGCGGATCGTTGAGAACA ACAACCAGCGCGAAGC§	TM-MAGE
ftsI	Mut1ftsIwt_F	ACCCCGGTCAGGGTTAATTC	Mutant screening PCR
ftsI	Mut1ftsIM_F	ACCCCGGTCAGGGTTAATTT	Mutant screening PCR
ftsI	Mut1ftsI_R	ACCATCACCGACGTGTTTGA	Mutant screening PCR
ftsI	Mut2ftsIwt_F	CTTCGCGCTGGTTGTTGTTA	Mutant screening PCR
ftsI	Mut2ftsIM_F	CTTCGCGCTGGTTGTTGTTC	Mutant screening PCR
ftsI	Mut2ftsI_R	CCTGTCCCCTCGCCTTGATT	Mutant screening PCR
ftsI	Mut1ftsIwt2_F	CGTGGCCGTTAATTCGATAA	Mutant screening PCR
ftsI	Mut1ftsIM2_F	CGTGGCCGTTAATTCGATAG	Mutant screening PCR
ftsI	Mut1ftsI2_R	CGAAAGAGGCGATGCGTAAC	Mutant screening PCR
ompC	ompC_Xba_F	*CAGAATCTAGA*GTTTTGACATTCAGTG CTGTCA	Cloning in pACY184
ompC	ompC_Hind_R	*AACATAAGCTT*TCATGCGAACGGTCGC AAGA	Cloning in pACY184
ompC	ompC_F2	CAGACATTCAGAAATGAATGACGGT	Sequence verification
ompC	ompC_R2	AGTGCCACGCGGGTAAAGCT	Sequence verification
ompC	ompC_St38_F	TAGTGCTCATGGCGAAGGTA	Sequence verification
ompC	ompC_St38_R	GTACCGATCAGGCCAGTGT	Sequence verification
pACY184	pACY184_F	AAGAGATTACGCGCAGACCA	Sequence verification
pACY184	pACY184_R	ATAGTGACTGGCGATGCTGT	Sequence verification

§ * indicates a phosphorothioate bonds

Table S4: Mutations detected in MG1655 strains derivatives mutated in *ftsI*.

ftsI mutation	Mutant name	Position	Mutation	Gene affected	*Effect
Control§	Magect_1	1,64,569	A=>G	*ydfA*	NS
		1,842,545	C=>T	*gdhA*	NS
		2,236,418	G=>A	*preA*	NS
		2,426,060	A=>G	*hisJ*	S
YRIN	Mage_1a	92,411	Ins 12nt	*ftsI*	**YRIN**
		777,276	$(G)_{6\rightarrow7}$	*tolA*	FS
		979,184	C=>T	*mukB*	S
		4,261,075	G=>T	*yjbM*	NS
E349K	Mage_2b	92,457	G=>A	*ftsI*	**E349K**
		380,022	$(G)_{10\rightarrow9}$	*frmR*	IG
		1,475,884	A=>G	*ydbD*	S
I532L	Mage_3c	93,006	A=>C	*ftsI*	**I532L**
YRIN+E349K	Mage_4b	92,411	Ins 12nt	*ftsI*	**YRIN**
		92,457	G=>A	*ftsI*	**E349K**
E349K + I532L	Mage_5a	92,457	G=>A	*ftsI*	**E349K**
		93,006	A=>C	*ftsI*	**I532L**
		21,878	T=>C	*ribF*	NS
		2,958,079	A=>G	*ptrA*	NS
YRIN+E349K + I532L	Mage_6b	92,411	Ins 12nt	*ftsI*	**YRIN**
		92,457	G=>A	*ftsI*	**E349K**
		93,006	A=>C	*ftsI*	**I532L**
		994,214	C=>A	*ssuC*	NS

§Control strain was obtained by submitting the MGF strain to TM-MAGE steps but without incorporating mutations in *ftsI*. §NS, non-synonymous, S, synonymous; FS Frameshift, IG intergenic. Mutations resulting of site directed mutagenesis are indicated in red

Table S5: Genomic features of the *E. coli* ST410 isolate *Ec*-MAD

	Replicon	Size (bp)	Number of CDS	Antibiotic resistance genes
Chromosome		4,747,851	4,452	$bla_{\text{CMY-2}}$
p*Ec*MAD1	IncFIB, IncFIA, IncQ1	98,473	15	*dfrA17, mph(A), aadA5, sul1, tet(B), aac(3)-IId, aac(6')Ib-cr,* $bla_{\text{OXA-1}}$, $bla_{\text{CTX-M-15}}$, $bla_{\text{TEM-1B}}$, *sul2, strA, strB*
p*Ec*MAD2	IncX3, ΔColKP3	51,479	65	*qnrS1*, $bla_{\text{OXA-181}}$
p*Ec*MAD3	Col (BS512)	2,088	2	-

Table S6 Antibiotics susceptibility testing of the ST410 isolate *Ec*-MAD

Resistant	Amoxicillin, Ticarcillin, Piperacillin, Cefepime, Cefalotin, Amoxicillin-Clavulanate, Ceftazidime, Piperacillin-Tazobactam, Cefoxitin, Cefuroxime, Ticarcillin-Clavulanate, Aztreonam, Moxalactam, Cefotaxime, Streptomycin, Clarythromycin, Tetracycline, Erythromycin, Rifampicin, Ciprofloxacin, Nalidixic Acid, Trimethoprim, Tobramycin
Intermediate	Mecillinam, Ertapenem, Kanamycin and Gentamicin
Susceptible	Imipenem, Meropenem, Doripenem, Amikacin, Azithromycin, Chloramphenicol, Tigecycline and Colistin

Table S7. Distribution and effect of point mutations in the OXA-181 *E. coli* ST410 subclade MRCA

**Region**	**SNPs**	**Synonymous**	**Non-Synonymous**	**Start/Stop**	**Functional SNP***	**IG§**
Non-recombinant	84	23	46	2	25	11
Recombinant&	1622 (197)	1303 (169)	205 (16)	2	9 (1)	112 (11)

*As determined by the SIFT algorithm[5], §Intergenic regions, &In bracket the number of SNPs in the 16.5 kb region of the *dcw* gene cluster common to the five recombinant regions in *Ec* ST410 isolates.

Table S8. Mutations predicted with a functional effect using the SIFT algorithm

Locus Tag	Gene	Product	Mutation	SIFT Score*	Median Info[&]	Chromosomal region	Functional class
EcMAD_00039	*caiD*	crotonobetainyl-CoA hydratase	S181N	0.01	2.75	Non-recombinant	Nitrogen metabolism
EcMAD_00067	*yabI*	hypothetical protein	G254V	0	3.38	Recombinant	Conserved hypothetical
EcMAD_00068	*thiQ*	thiamin ABC transporter - ATP binding subunit	T66M	0.03	2.76	Recombinant	Transporter
EcMAD_00075	*setA*	sugar / lactose efflux transporter SetA	V27G	0.01	2.76	Recombinant	Transporter
EcMAD_00088	*ftsI*	essential cell division protein FtsI; penicillin-binding protein 3	I536L	0.02	2.75	Recombinant	Cell envelope
EcMAD_00134	*yadE*	putative polysaccharide deacetylase lipoprotein	P171S	0.01	2.77	Recombinant	Cell envelope
EcMAD_00143	*htrE*	putative outer membrane usher protein	V662I	0.05	2.75	Recombinant	Cell envelope
EcMAD_00152	*hrpB*	putative ATP-dependent helicase	C332S	0.01	2.76	Recombinant	DNA metabolism
EcMAD_00152	*hrpB*	putative ATP-dependent helicase	P553L	0.01	2.76	Recombinant	DNA metabolism
EcMAD_00155	*fhuC*	iron (III) hydroxamate ABC transporter - ATP binding subunit	T72A	0.03	2.77	Recombinant	Transporter
EcMAD_00362	*brnQ*	branched chain amino acid transporter BrnQ	D385N	0	2.77	Non-recombinant	Transporter
EcMAD_00854	*nfsA*	NADPH nitroreductase monomer	R203H	0	2.77	Non-recombinant	Stress response
EcMAD_00986	*rarA*	acid phosphatase	A300V	0	2.77	Non-recombinant	DNA metabolism
EcMAD_01114	*ymdA*	putative protein	I21S	0.03	2.83	Non-recombinant	Conserved hypothetical
EcMAD_01472	*tehA*	TehA TDT transporter	P246L	0	2.77	Non-recombinant	Transporter
EcMAD_01480	*hicB*	antitoxin of the HicA-HicB toxin-antitoxin system	T34A	0	2.75	Non-recombinant	Stress response
EcMAD_01535	*dosP*	c-di-GMP phosphodiesterase, heme-regulated	G524D	0.02	2.76	Non-recombinant	Stress response
EcMAD_01541	*yddB*	putative porin protein	G406C	0	2.78	Non-recombinant	Cell envelope
EcMAD_01796	*astE*	succinylglutamate desuccinylase	A45V	0.03	2.78	Non-recombinant	Carbon metabolism
EcMAD_01900	*edd*	phosphogluconate dehydratase	R312H	0.05	3.22	Non-recombinant	Carbon metabolism
EcMAD_02162	*yegV*	putative kinase	I181T	0	2.77	Non-recombinant	Conserved hypothetical
EcMAD_02201	*yohC*	putative inner membrane protein	L134Q	0	2.78	Non-recombinant	Conserved hypothetical
EcMAD_02264	*ccmB*	protoheme IX ABC transporter - membrane subunit CcmB	G147S	0	2.77	Non-recombinant	Transporter
EcMAD_02280	*ompC*	outer membrane porin C	R191L	0.03	3.14	Non-recombinant	Cell envelope
EcMAD_02311	*glpB*	glycerol-3-phosphate dehydrogenase, membrane anchor subunit	V289M	0.02	2.76	Non-recombinant	Carbon metabolism
EcMAD_02425	*emrK*	EmrKY-TolC multidrug efflux transport system - membrane protein	N248I	0	2.75	Non-recombinant	Transporter
EcMAD_02511	*maeB*	malate dehydrogenase	A172S	0.04	3.14	Non-recombinant	Carbon metabolism
EcMAD_02714	*nrdE*	ribonucleoside-diphosphate reductase 2	F84L	0.01	3.3	Non-recombinant	DNA metabolism
EcMAD_02760	*hycI*	hydrogenase 3 maturation protease	A63V	0.02	2.79	Non-recombinant	Carbon metabolism
EcMAD_02802	*casC*	Cascade subunit C	P125S	0	2.75	Non-recombinant	DNA metabolism
EcMAD_03071	*gss*	glutathionylspermidine amidase / glutathionylspermidine synthetase	P100S	0.01	2.81	Non-recombinant	Nitrogen metabolism
EcMAD_03159	*ebgR*	EbgR DNA-binding transcriptional repressor	I98T	0	2.77	Non-recombinant	C. metabolism Regulation
EcMAD_03286	*yrbG*	inner membrane protein YrbG	G219V	0.01	2.76	Non-recombinant	Conserved hypothetical
EcMAD_03840	*emrD*	multidrug efflux transporter EmrD	G323D	0	2.76	Non-recombinant	Transporter

* Ranges from 0 to 1, mutation is predicted to be functional for values equal or below 0.05. [&]Median for the whole alignment of the information content calculated for each position. This value ranges from 0, when all 20 amino acids are identified to 4.32 when only one amino acid is found.

Table S10. Antibiotic resistance gene content of ST410 isolates analysed in Fig. 7*

	*qnr*B4	*qnr*S1	*aac*(3)-IId	*aac*(6')Ib-cr	*aad*A5	*bla*CMY-2	*bla*CMY-42	*bla*CTX-M-15	*bla*DHA-1	*bla*OXA-1	*bla*OXA-181	*bla*TEM-1B	*dfr*A17	*mph*(A)	*strA*	*strB*	*Sul1*	*sul2*	*tet*(A)	*tet*(B)
83B9	+	+	+	+	+	+		+	+	+	+	+	+	+	+	+	+	+		+
*Ec*MAD		+	+	+	+	+		+		+	+	+	+	+	+	+	+	+		+
94G8		+			+		+	+			+		+	+			+	+	+	
92B7		+		+	+			+		+	+		+	+			+		+	
93G1		+		+	+			+		+	+		+	+			+		+	
32139			+	+	+			+		+		+	+	+	+	+	+	+		+

*β-lactamase genes are highlighted with colours; + indicates that the gene is present.
