# Supplementary material for: Stepwise evolution and convergent recombination underlie the global dissemination of carbapenemase-producing Escherichia coli
Source: Genome Med. 2020 Jan 20;12:10. doi: 10.1186/s13073-019-0699-6 (PMC6970295; doi:10.1186/s13073-019-0699-6)
Supplement: Supplementary file 4 — Additional file 4: Figure S1. Number of antibiotic resistance genes (ARG) according to the phylogenetic group among E. coli ST410 isolates. Figure S2. Recombination events in isolates of the E. coli ST410 FQR clade. Figure S3. Phylogenetic distribution of E. coli isolates mutated in ftsI. Figure S4. Recombination events at the dcw and ompC loci. Figure S5. Phylogeny and mutations in non-redundant E. coli ST131 CP-Ec isolates. Figure S6. Phylogeny and mutations in non-redundant CP-Ec isolates of ST48, ST206, ST224, ST359, ST361, ST448 and ST617. Figure S7. Phylogeny and mutations in non-redundant E. coli isolates of phylogroup D. Figure S8. Phylogeny and mutations of ST10, ST226, and ST746 non-redundant E. coli isolates. Figure S9. Estimated fitness of E. coli ST410 strains in rich and minimal media. [file 13073_2019_699_MOESM4_ESM.pdf]

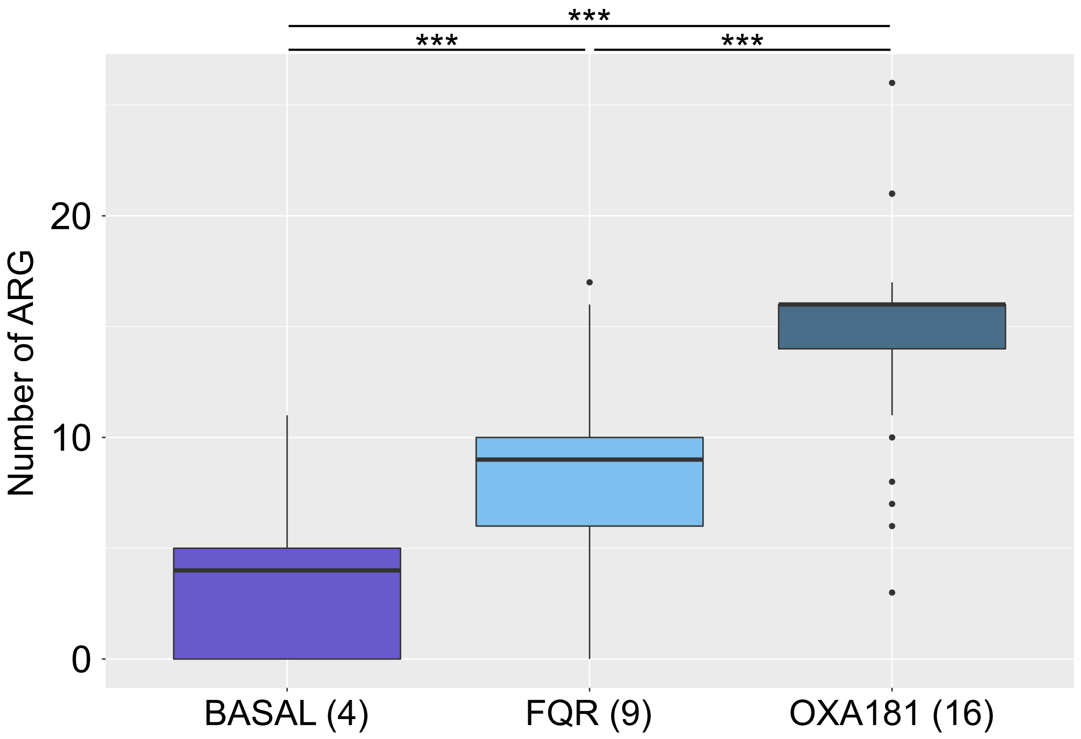

Figure S1 Number of antibiotic resistance genes (ARG) according to the phylogenetic group among *E. coli* ST410 isolates. The horizontal lines in the boxes represent the median number of ARG associated to the three clades: Basal (n=22); FQR clade (n=92) and OXA-181 subclade (n=41). The box boundaries represent the first and third quartiles of the distribution and box-plot whiskers span 1.5 times the interquartile range of the distribution. Outliers are denoted as black dots outside whiskers. Statistical significances were tested with a one-sided Wilcoxon rank-sum test. ***, $P < 0.001$.

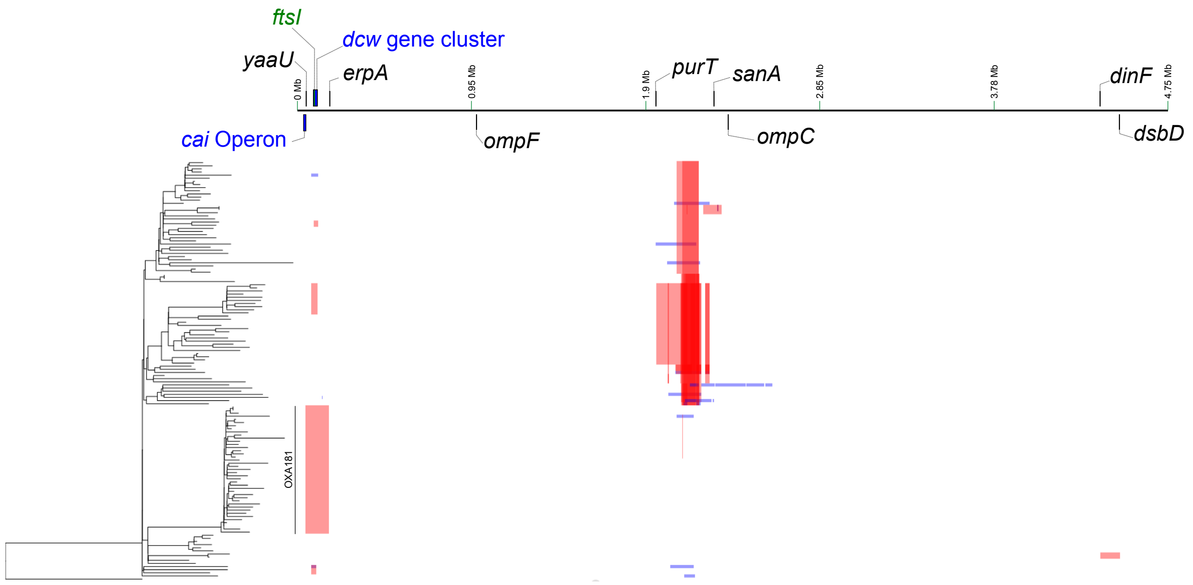

Figure S2. Recombination events in isolates of the *E. coli* ST410 FQR clade. Phylogeny of the *E. coli* ST410 FQR clade (left). On the right side, the recombined blocks identified by Gubbins [1] in the core genome, are highlighted in red when they occurred in an internal branch and affect more than one isolate and in blue when they occurred in a terminal branch affecting a single isolate. Only recombination events occurring within the FQR clade are represented. The genome of *E. coli* ST410 FQR strains is represented above the figure and annotated genes correspond to the limits of major recombination events.

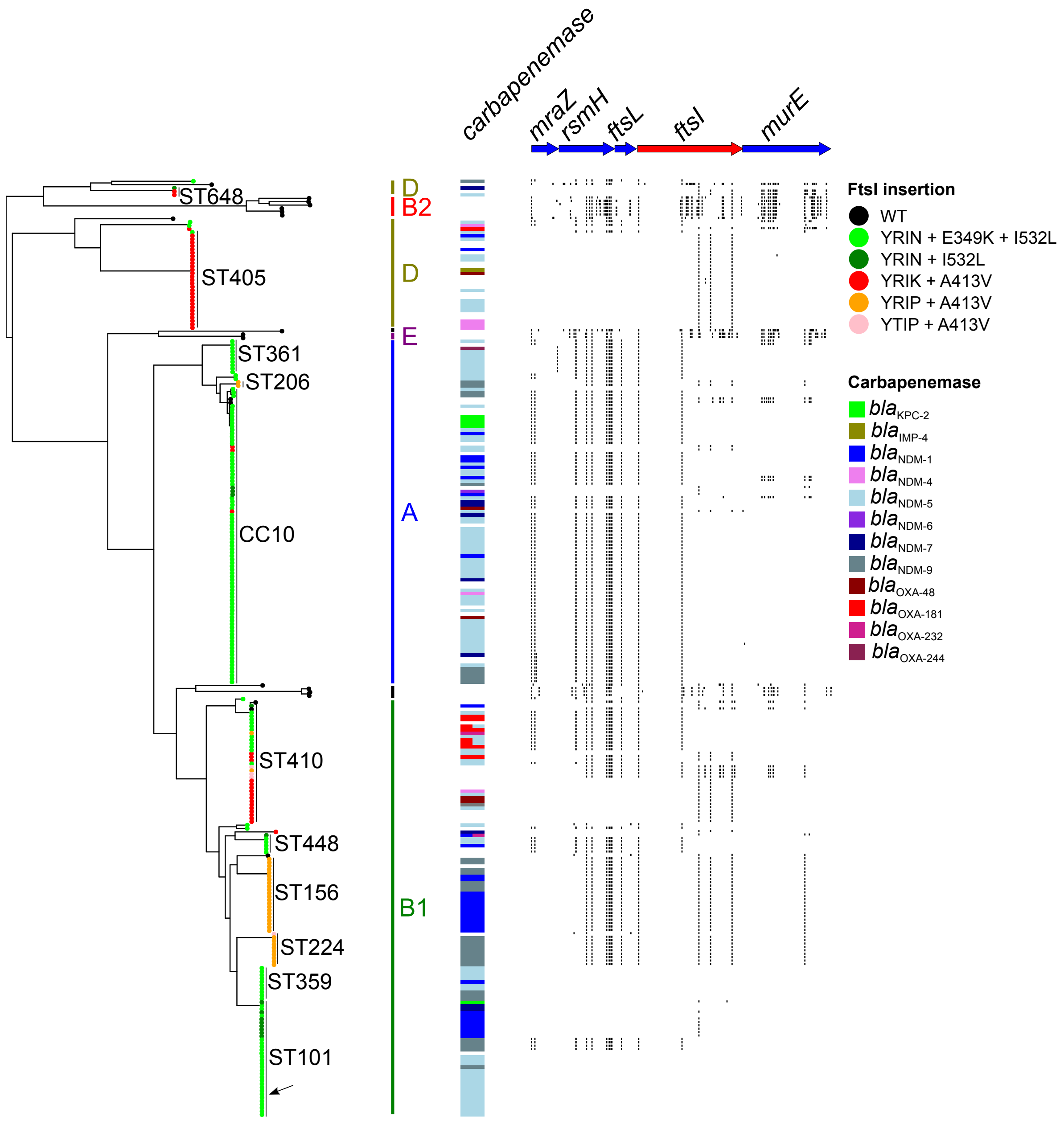

Figure S3. Phylogenetic distribution of *E. coli* isolates mutated in *ftsI*. The genome based phylogeny of *Ec* isolates mutated in *ftsI* and of *E. coli* reference strains[Touchon, 2009 #1177] was estimated from core non-recombinant SNP. Mutations in the *ftsI* gene are indicated at the tip of the branch as defined in the figure keys. Major STs are indicated, as well as phylogroups, S standing for *Shigella*. Carbapenemase genes are color-coded as defined in the figure keys on the right. SNPs in the *mraZ* – *murE* region compared to the *Ec* ST101 strain CREC-591 (CP024821) shown by a black arrow are indicated by small vertical black bars. An ST101 isolate was chosen as reference, as insertion of YRIN in *ftsI* occurred among this ST. Conservation of SNP patterns between isolates showing the same mutation in *ftsI* across the *E. coli* species revealed the exchange of these alleles by recombination.

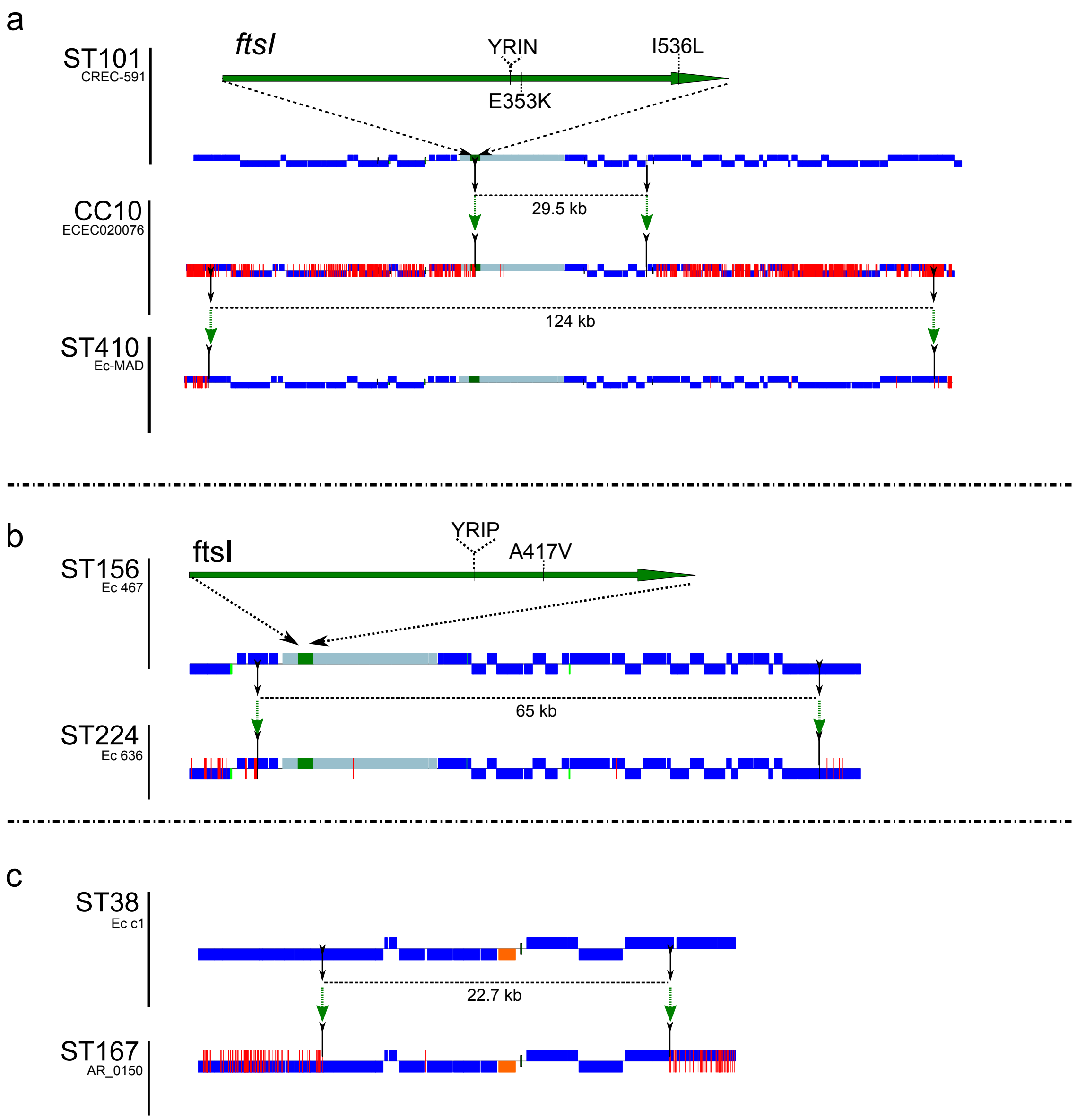

Figure S4. Recombination events at the *dcw* and *ompC* loci. a. Recombination of the *dcw* cluster encompassing *ftsI* carrying an YRIN insertion. The *ftsI* gene (in green) of an *E. coli* ST101 isolate was affected by a 12-nucleotide duplication leading to an YRIN insertion and by two additional mutations (E349K and I532L). A 29.5 kb chromosomal region of an ST101 isolate mutated in *ftsI* comprising most of the *dcw* gene cluster was transferred by recombination into a CC10 strain, which transferred a 130-kb fragment containing the *dcw* locus to the common ancestor of the OXA-181 ST410 subclade. **b.** Recombination of *ftsI* carrying an YRIP insertion. A 65-kb region from an *Ec* ST156 isolate affected by YRIP duplication and A413V mutation was transferred by recombination into an *E. coli* ST224 strain. **c.** Recombination of *ompC* gene from an ST38 strain. A 22.7-kb region encompassing the *ompC* gene (in orange) was transferred by recombination into the MRCA genome from the ST167 clade of isolates expressing different carbapenemase genes (Supplementary fig. 3). Small vertical red bars represent SNPs compared to the proposed donor strains as indicated on the right of the figure. Vertical black arrows indicate the limits of the recombined regions and green arrows the direction of exchange. CDSs are indicated by blocks, upper line transcribed in the rightward orientation and lower line in the opposite orientation. The *dcw* gene cluster is in grey.

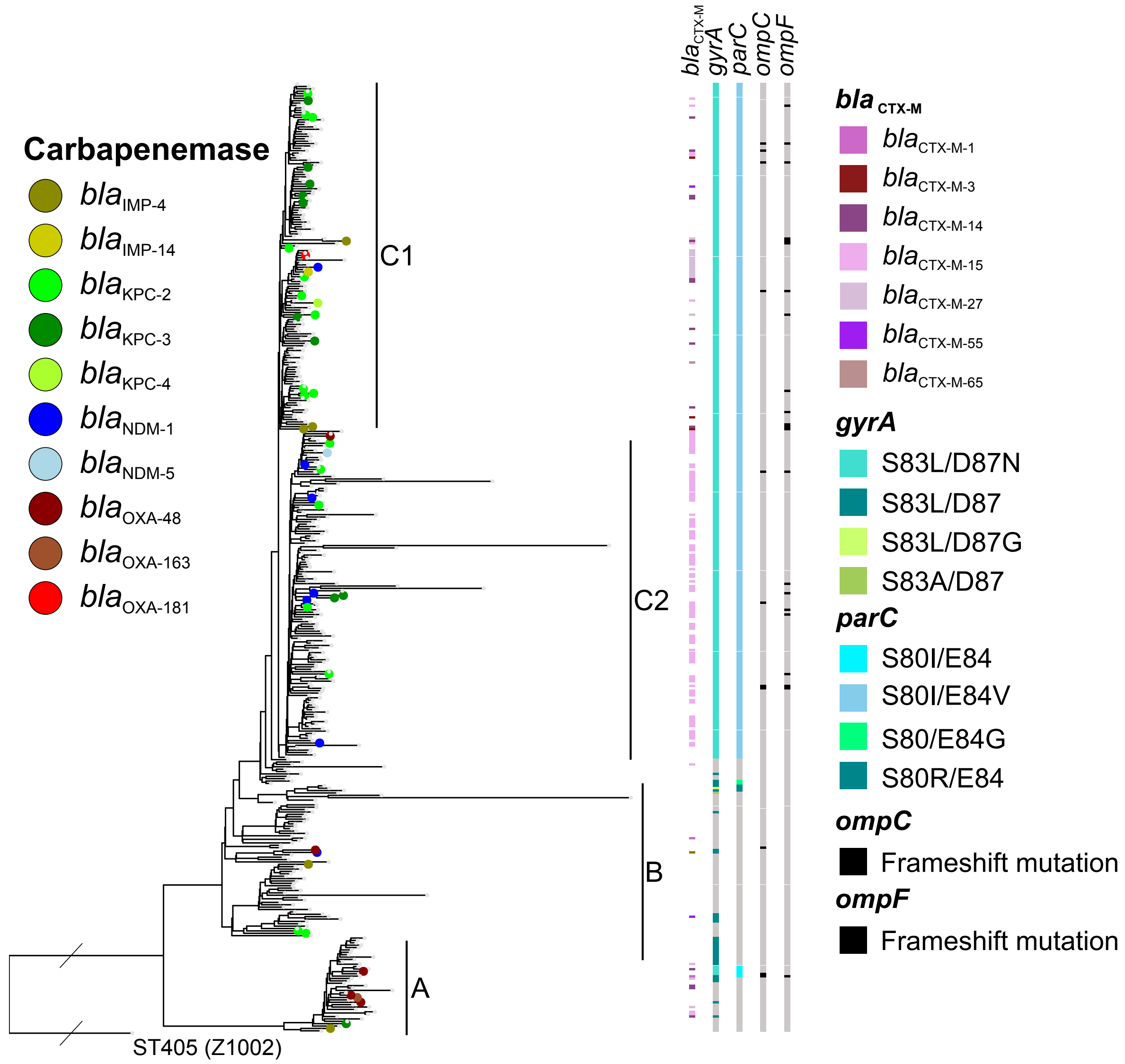

Figure S5. Phylogeny and mutations in non-redundant *E. coli* ST131 CP-*Ec* isolates. Maximum likelihood phylogeny was obtained from the alignment of 62,886 non-recombinant SNPs. Tree was rooted with the ST405 strain Z1002 as an outgroup. Vertical lines represent ST131 clades (A, B, C1, C2) as previously described[2]. Branch tips represent the carbapenemase (or absence) type as defined in the figure key on the left. Columns represent, from left to right: bla_{CTX-M} ESBL type, mutations in the QRDR regions of *gyrA* and *parC*, and mutations inactivating *ompC* and *ompF* porin genes as defined in the figure key on the right.

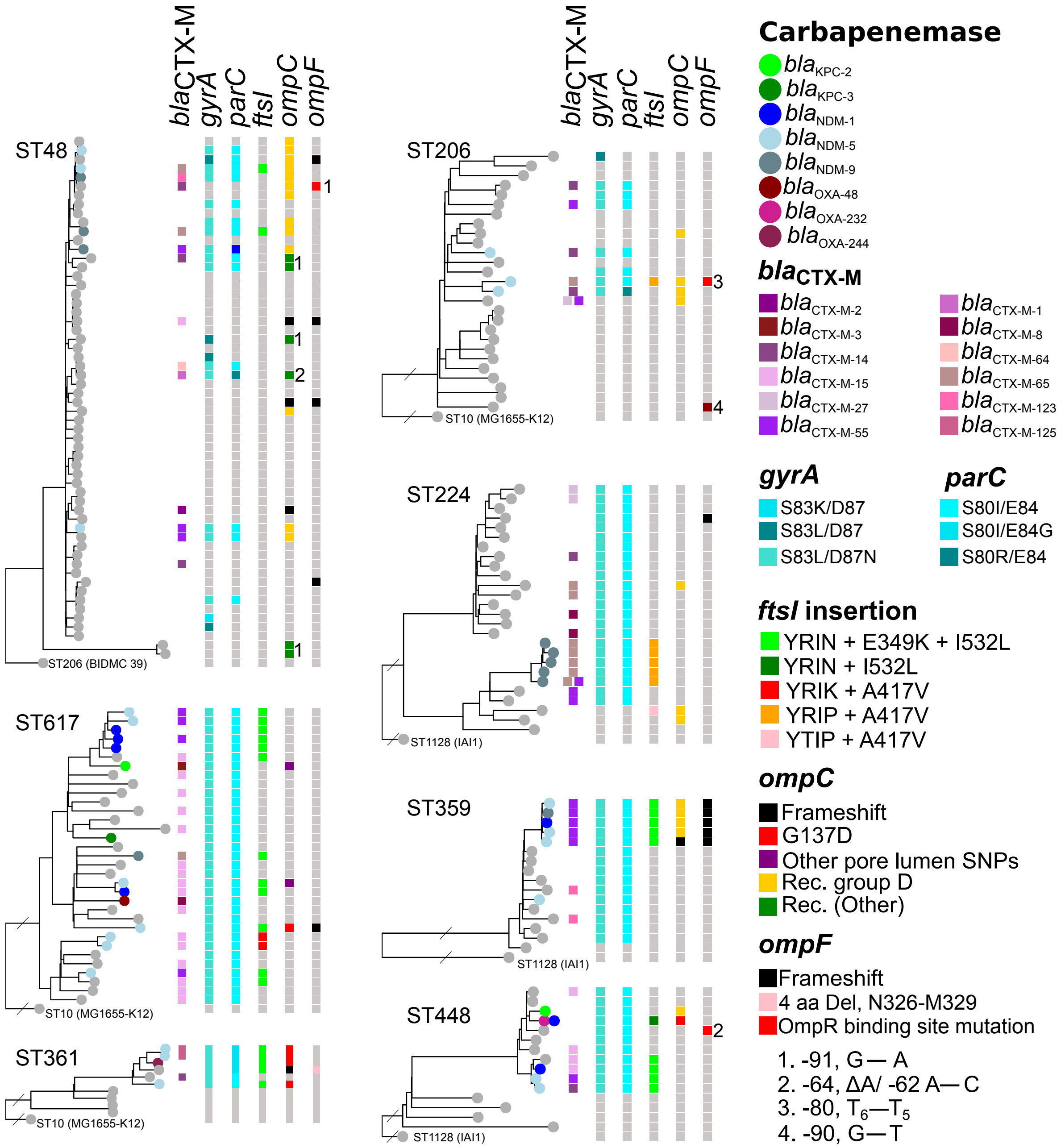

Figure S6. Phylogeny and mutations in non-redundant CP-*Ec* isolates of ST48, ST206, ST224, ST359, ST361, ST448 and ST617. Maximum likelihood phylogenies were estimated from 9,909 (ST48), 10,326 (ST206), 4,953 (ST224), 16,239 (ST359), 18,424 (ST361), 15,469 (ST448), and 2,093 (ST617) core non-recombinant SNPs. Isolates used to root the trees are at the bottom of each tree. Branch tips represent the carbapenemase (or absence) type. Columns on the right side of the trees represent from left to right: *bla*CTX-M ESBL type, mutations in *gyrA* and *parC* QRDR and in *ftsI* and genetic events affecting *ompC* and *ompF* according to the figure key on the right. When different alleles of *ompC* where acquired by recombination within the same ST, they are numbered.

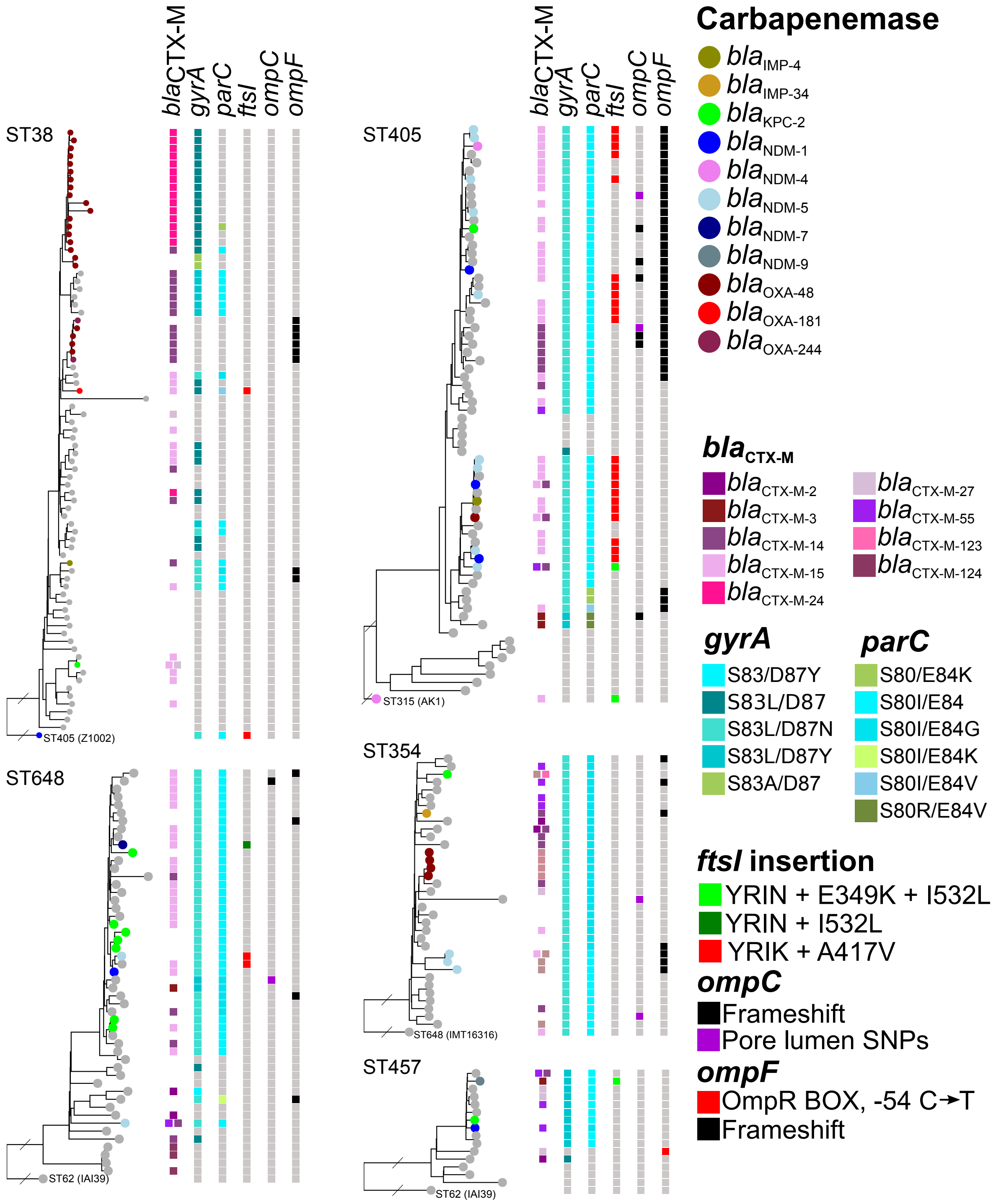

Figure S7 Phylogeny and mutations in non-redundant *E. coli* isolates of phylogroup D. Maximum likelihood phylogeny of phylogroup D isolates of ST38, ST354, ST405, ST457 and ST648 were obtained from 29,413, 35,955, 3,481, 32,603, and 19,924 core non-recombinant SNPs respectively. Isolates used to root the trees are at the bottom of each tree. Branch tips represent the carbapenemase type encoded or the absence of a carbapenemase according to the figure key. The columns represent, from left to right: *bla*$_{CTX-M}$ ESBL type, mutations in *gyrA* and *parC* QRDR, *ompC* and *ompF* according to the figure key.

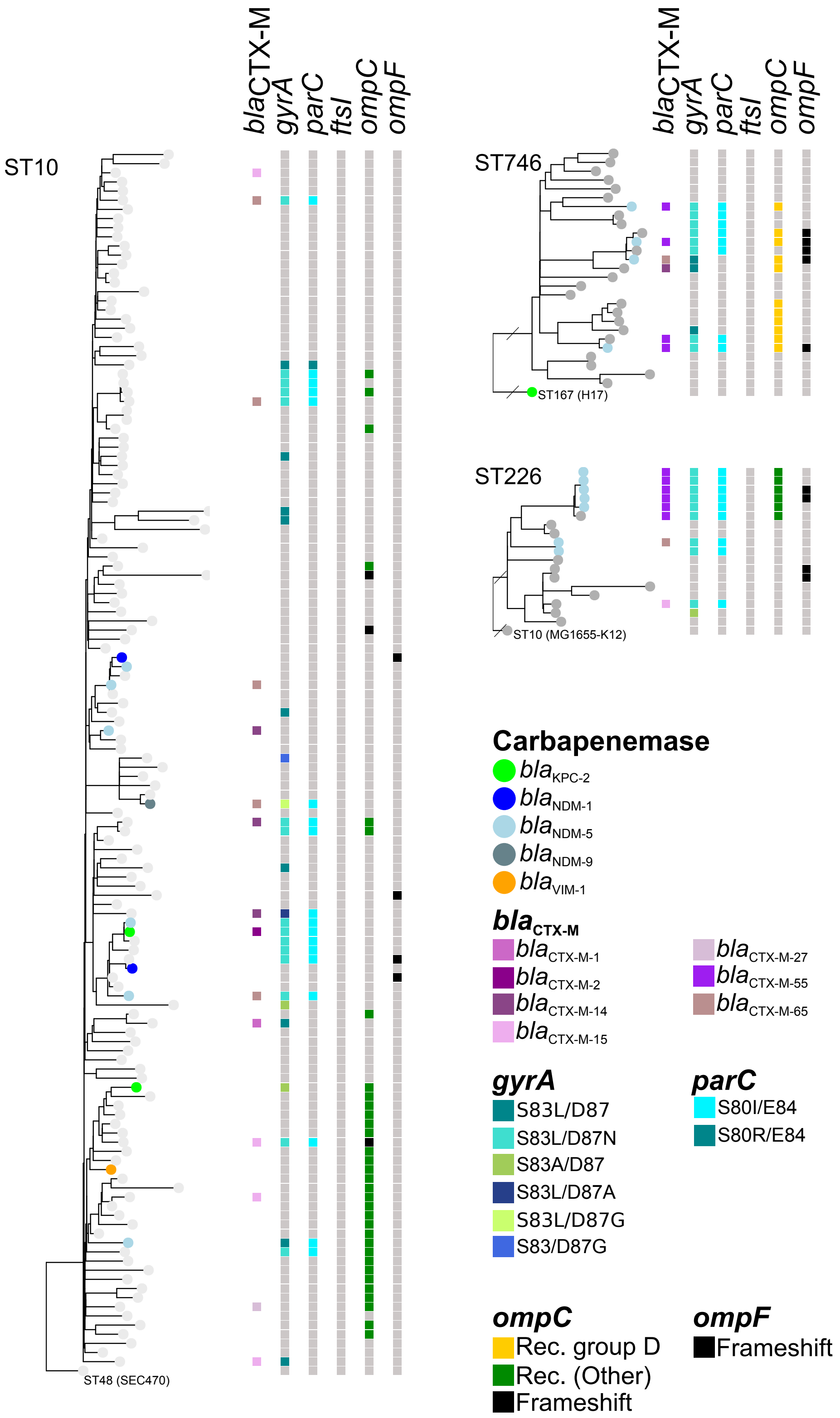

Figure S8 Phylogeny and mutations of ST10, ST226, and ST746 non-redundant *E. coli* isolates. Maximum likelihood phylogenies of ST10, ST226, and ST746 were estimated from 9,981, 6,635, and 44,664 core non-recombinant SNPs respectively. Isolates used to root the trees are at the bottom of each tree. In the ST10 final alignment the number of sequences was reduced from 765 to 137 by clustering sequences of non CP-*Ec* isolates with an identity over 99 % using cd-hit[3]. Branch tips represent the carbapenemase (or absence) type as defined in the figure key. Columns represent, from left to right: *bla*$_{\text{CTX-M}}$ ESBL type, mutations in *gyrA* and *parC* QRDR, *ompC* and *ompF* according to the figure key on the right.

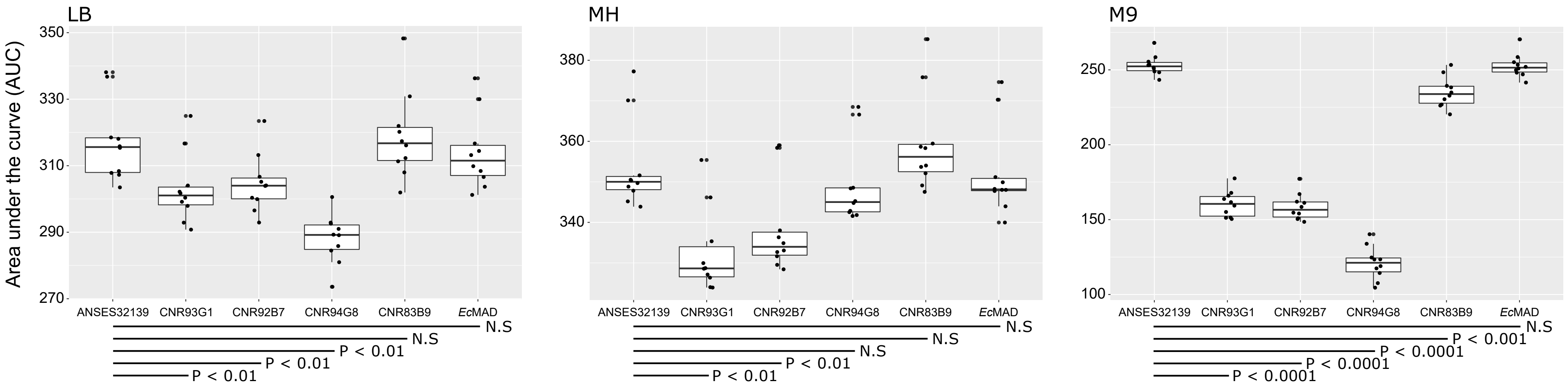

Figure S9. Estimated fitness of *E. coli* ST410 strains in rich and minimal media. Fitness was estimated based on area under the curve of ten replicates determined by using growthcurver[4]. The box boundaries represent the first and third quartiles of the distribution and box-plot whiskers span 1.5 times the interquartile range of the distribution. Outliers are denoted as dots outside whiskers. Statistical significances were tested with a Wilcoxon two-sided test comparing the values of all strains against strain ANSES32139, which does not encode the *bla*$_{\text{OXA-181}}$ carbapenemase.

References

1. Croucher NJ, Page AJ, Connor TR, Delaney AJ, Keane JA, Bentley SD, Parkhill J, Harris SR: Rapid phylogenetic analysis of large samples of recombinant bacterial whole genome sequences using Gubbins. *Nucleic Acids Res* 2015, 43:e15.
2. Stoesser N, Sheppard AE, Pankhurst L, De Maio N, Moore CE, Sebra R, Turner P, Anson LW, Kasarskis A, Batty EM, et al: Evolutionary History of the Global Emergence of the *Escherichia coli* Epidemic Clone ST131. *MBio* 2016, 7:e02162.
3. Li W, Godzik A: Cd-hit: a fast program for clustering and comparing large sets of protein or nucleotide sequences. *Bioinformatics* 2006, 22:1658-1659.
4. Sprouffske K, Wagner A: Growthcurver: an R package for obtaining interpretable metrics from microbial growth curves. *BMC Bioinformatics* 2016, 17:172.
